# Incidence of Skeletal-Related Events in Patients with Castration-Resistant Prostate Cancer: An Observational Retrospective Cohort Study in the US

**DOI:** 10.1155/2019/5971615

**Published:** 2019-07-09

**Authors:** Alison Tse Kawai, David Martinez, Catherine W. Saltus, Zdravko P. Vassilev, Montse Soriano-Gabarró, James A. Kaye

**Affiliations:** ^1^RTI Health Solutions, Waltham, Massachusetts, USA; ^2^RTI Health Solutions, Barcelona, Spain; ^3^Bayer US, Whippany, New Jersey, USA; ^4^Bayer AG, Berlin, Germany

## Abstract

**Background and Objective:**

Skeletal-related events (SREs) are common in men with bone metastases and have negative consequences for patients with castration-resistant prostate cancer (CRPC), including pain, reduced quality of life, and increased mortality. We estimated incidence rates of first SREs in a cohort of men with CRPC in the Surveillance, Epidemiology, and End Results-Medicare database.

**Methods:**

We included men aged ≥ 65 years with a prostate cancer diagnosis in 2000-2011 if they had no prior malignancy (other than nonmelanoma skin cancer) and had surgical or medical castration with subsequent second-line systemic therapy, which was used to infer castration resistance. The first occurrence of an SRE (fracture, bone surgery, radiation therapy, or spinal cord compression) in Medicare claims was identified. Incidence rates of SREs were estimated in all eligible person-time and, in secondary analyses, stratified by any use of bone-targeted agents (BTAs) and history of SRE.

**Results:**

Of 2,234 men with CRPC (84% white, mean age = 76.6 years), 896 (40%) had an SRE during follow-up, with 74% occurring within a year after cohort entry. Overall, the incidence rate of SREs was 3.78 (95% CI, 3.53-4.03) per 100 person-months. The incidence rate of SREs before any BTA use was 4.16 (95% CI, 3.71-4.65) per 100 person-months, and after any BTA use was 3.60 (95% CI, 3.32-3.91) per 100 person-months. The incidence rate in patients with no history of SRE was 3.33 (95% CI 3.01-3.68) per 100 person-months, and in patients who had such a history, it was 4.20 (95% CI 3.84-4.58) per 100 person-months.

**Conclusions:**

In this large cohort of elderly men with CRPC in the US, SREs were common. A decrease in incidence of SREs after starting BTA is suggested, but the magnitude of the effect may be confounded by indication and other factors such as age and prior SRE.

## 1. Introduction

Skeletal-related events (SREs) are common in men with bone metastases and have negative consequences for patients with castration-resistant prostate cancer (CRPC), including pain, reduced quality of life, and increased risk of death [1, 2]. Studies have examined the incidence of SREs in cohorts of men with metastatic prostate cancer [1, 3–5], but no population-based studies have been conducted in men with prostate cancer starting at the time of castration resistance. The objective of this study was to estimate incidence rates of SREs in a cohort of men with CRPC in the Surveillance, Epidemiology, and End Results (SEER)-Medicare-linked database. The SEER-Medicare–linked database is the largest available source of detailed population-based medical information on men age 65 years and older with prostate cancer [6]. 

## 2. Methods

### 2.1. Study Population

The present study used a retrospective, observational cohort of men with CRPC identified in SEER-Medicare data that was used previously to estimate incidence rates of second primary malignancies [7]. In brief, the cohort consisted of men age 65 years and older with a prostate cancer diagnosis in 2000-2011 in SEER data who met the protocol-defined criteria for castration resistance: surgical or medical castration, followed by initiation of second-line systemic therapy, from which castration resistance was inferred (see Saltus et al. [8] for further details on how CRPC was identified). Included were men who had no prior diagnoses of other cancers (other than prostate cancer or nonmelanoma skin cancer) or metastases (other than bone or lymph node) on or before the date of initiation of second-line systemic therapy. Patients were also required to be enrolled in Medicare Parts A and B continuously from the date of prostate cancer diagnosis or from 1 year before the initiation of second-line systemic therapy (whichever occurred first) to the initiation of second-line systemic therapy and could not be enrolled in a health maintenance organization during the year before initiation of second-line systemic therapy.

### 2.2. Outcomes

The primary outcome, SRE, was defined as the first occurrence of the following during follow-up: fracture, bone surgery, radiation, or spinal cord compression and was identified in Medicare claims data using diagnosis and procedure codes listed in Supplementary Tables 1-2. Secondary outcomes included identifying (1) whether the first-occurring SRE was a fracture (either pathologic or traumatic fracture) and (2) whether the first-occurring SRE was a pathologic fracture. The first occurrence of an SRE during follow-up was ascertained from the date that CRPC was identified (i.e., date of initiation of systemic second-line therapy) to the earliest of the following: death, second primary malignancy, discontinuation of Part A or Part B Medicare coverage, enrollment in a health maintenance organization, or the end of Medicare data availability at the time of the study (December 31, 2013).

### 2.3. Analysis

Incidence rates of SREs (per 100 person-months) and 95% confidence intervals (CIs) were calculated in all person-time following cohort entry. In secondary analysis, we also estimated incidence rates stratified separately by (1) history of SRE prior to cohort entry and (2) any use of the following bone-targeted agents (BTAs) after the prostate cancer diagnosis: alendronate, denosumab, ibandronate, pamidronate, risedronate, or zoledronic acid.

For the analysis stratified by any use of BTAs, we first identified whether patients had any BTA use after the initial prostate cancer diagnosis. In patients with no BTA use after the initial prostate cancer diagnosis, all person-time was classified as “before any BTA use.” In patients with BTA use between the prostate cancer diagnosis and cohort entry (i.e., identification of castration resistance), all person-time was classified as “after any BTA use.” Patients who initiated BTA use after cohort entry contributed person-time to both the “before any BTA use” and “after any BTA use” strata.

## 3. Results

In the SEER-Medicare data set, 564,491 men were diagnosed with prostate cancer during the study period. Of those, 2,234 individuals met the protocol-defined criteria for CRPC and other eligibility criteria for the cohort. The cohort was primarily white (83.6%), and mean age at cohort entry was 76.6 years (Table 1). More than half (56%) of the cohort had a history of SRE prior to cohort entry. Bone-targeted agent use in the cohort was common, with initial use before cohort entry in 54.3% of patients and initial use after cohort entry in 14.6% of patients. Mean (SD) follow-up was 10.6 (11.6) months, with 30.6% of patients contributing more than 1 year of follow-up.

SREs after development of castration resistance were common, with 40% of the cohort experiencing an SRE during follow-up. Patients with SREs during follow-up tended to be younger than those without SREs (Table 2). Mean (SD) time from cohort entry to first SRE during follow-up was 9 (10.1) months. More than half of patients (74.3%) had their first SRE within a year after cohort entry, 18.3% during the second year, and 7.4% more than 2 years after cohort entry.

Table 3 shows the cumulative incidence of first SRE during follow-up by event type (fracture, bone surgery, radiation, and spinal cord compression). The most common types of SREs were identified as use of radiation therapy (occurring in 27.3% of the cohort) and occurrence of a fracture (11.9% of the cohort). With respect to the type of event stratified by race, 12.6% of white patients and 6.9% of black patients had a fracture on the day of their first SRE. The percentages of patients with radiation therapy on the day of their first SRE were 27.7% among whites and 24.8% among blacks. Numbers of other first SREs (bone surgery and spinal cord compression) were too small to report stratified by race.

The incidence rate of first SRE during all person-time after cohort entry was 3.78 per 100 person-months (95% CI, 3.53-4.03) (Table 4). The incidence rate of SRE was slightly numerically lower following any use of BTAs compared with person-time before any use and numerically higher among patients with a history of SRE compared with patients who did not have such a history. When the number of first SREs was stratified by race, 769 first SREs were found in 19,390 person-months of follow-up among white men and 72 first SREs were found in 2,788 months of follow-up among black men. The incidence rate of first SRE for whites was 3.97 (95% CI, 3.69-4.26) and for blacks was 2.58 (95% CI, 2.02-3.25) per 100 person-months.

A total of 363 cases of fracture (pathologic or traumatic fracture) occurred during 31,257 person-months of follow-up, yielding an incidence rate of 1.16 per 100 person-months (95% CI, 1.04-1.29). A total of 176 cases of pathologic fracture occurred during 33,635 person-months of follow-up, for an incidence rate of 0.52 per 100 person-months (95% CI, 0.45-0.61).

In a Poisson regression model, we found a strong linear trend for a decrease in the incidence rate of first SRE with increasing duration of ADT before cohort entry. The incidence rate ratio (with years of ADT entered as a continuous independent variable) was 0.93 (95% CI, 0.90-0.96; P<0.0001), indicating a 7% decrease (95% CI, 4%-10%) in the incidence rate of first SRE with each additional year of ADT before cohort entry.  Table 5 shows the incidence rates of first SREs stratified by duration of ADT.

## 4. Discussion

In a cohort of 2,234 patients with CRPC identified in SEER-Medicare data with a mean follow-up of 10.6 months, the cumulative incidence of first SREs after development of castration resistance was 40%. The incidence rate of SREs was 3.78 per 100 person-months, reflecting a substantial effect of impaired bone health in men with CRPC.

To our knowledge, no observational studies have estimated incidence rates of SREs in men with CRPC starting at the time of castration resistance. An observational study of men who died from or were treated palliatively for metastatic CRPC at three medical centers in Canada reported an incidence rate of symptomatic skeletal events of 85.4 per 100 patient-years (7.1 per 100 person-months), which is higher than the incidence rate of SREs observed in the present analysis [3]. Notable differences from our study include that their study used medical records to assess multiple symptomatic skeletal events from the time of diagnosis of bone metastases until death. Further, the inclusion of patients who died from metastatic CRPC may have overestimated the incidence of skeletal events in the overall population of patients with metastatic CRPC. By contrast, our population-based cohort study of men with CRPC used claims algorithms (without medical record review) to assess the first occurrence of an SRE from the time that castration resistance was identified until end of study period, death, or second primary malignancy.

Other studies have examined the cumulative incidence of SREs in cohorts of men with metastatic prostate cancer. Compared with our estimate of the incidence of SREs in the cohort of men with CRPC identified in SEER-Medicare data (40%), the cumulative incidence was similar in a small cohort of men with bone metastatic CRPC identified from two Veterans Administration Medical Centers (38%) [4] and in another cohort of men with prostate cancer and bone metastases identified in SEER-Medicare data (40%) [1] but higher in cohorts of men with prostate cancer and bone metastases identified in the Danish National Patient Registry (1-year cumulative incidence of 46%) and in the Thomson MedStatMarketScan Commercial Claims and Encounter database (overall cumulative incidence of 53%) [5, 9]. The differences in estimates of cumulative incidence of SREs in those studies versus ours could be due to differences in data sources, outcome definitions, length of follow-up, and/or study populations. Notably, our cohort included men with CRPC regardless of whether they were known to have bone metastasis, although estimates in the literature report that approximately 90% of patients with metastatic prostate cancer have bone metastasis [10, 11]. As reported previously, 80.4% of our cohort of men with CRPC identified in SEER-Medicare data had claims associated with codes for bone metastases, and 84.5% either had claims for bone metastases or received bone-directed therapy [8]. However, as NCI states, no algorithm accurately and completely identifies metastases in SEER-Medicare claims [12]; because these codes may have suboptimal sensitivity, this may be an underestimate of the prevalence of bone metastases in the cohort [8].

We found a higher incidence rate of first SRE for white men than for black men. While the disease course for many cancers, including prostate cancer, is often found to be more accelerated and results in poorer outcomes for black men compared with their white counterparts [13–16], it is possible that higher bone density among black men [17, 18] may be associated with the lower rate of first SREs and the lower proportion of fractures among blacks observed here. A similar result was observed in a SEER-Medicare population of men with incident metastatic prostate cancer [19]. Our study cohort comprised such small proportions of men who were Asian, Hispanic, or of other/unknown race (<10% collectively) that we could not analyze their SRE counts or incidence rates further (since the data use agreement for using the SEER-Medicare linked data prohibit publication of cell counts <11).

The incidence rate of SRE was numerically higher among patients with a history of SRE compared with patients who did not have such a history and slightly numerically lower following any use of BTAs compared with person-time before any use. However, we did not estimate any measures of association or conduct formal hypothesis testing. Because this was an observational study with no adjustment for differences in patient characteristics (e.g., severity of disease and performance status) between subgroups, any comparison of rates before and after initial use of a BTA could be biased from confounding by indication, as patients at greater risk for an SRE are more likely to be prescribed a BTA. Information on such factors is not available in claims data. Further, the analysis of person-time stratified before and after initial use of BTAs did not account for dose, duration of use, or timing of use relative to diagnosis of prostate cancer or castration resistance.

We investigated whether the duration of ADT was associated with the incidence of first SRE and observed a decreasing rate of SREs with increasing duration of ADT. While one might hypothesize that longer duration of ADT would result in a higher rate of SREs because ADT can cause a decrease in bone density [20], ADT duration may be an indicator of the biological aggressiveness of prostate cancer in an individual patient (i.e., longer duration of ADT would be expected in a patient with more indolent prostate cancer). These results are consistent with the hypothesis that a longer duration of ADT predicts a lower rate of SREs, perhaps because longer duration of ADT is a marker for more indolent prostate cancer. The results do not support the notion that the rate of first SRE increases with duration of ADT (which might have been hypothesized to be mediated by a reduction in bone density).

The primary strength of this study is the use of the SEER-Medicare database, which is representative of the elderly population in the US and the largest available source of detailed population-based medical information on men aged 65 years or older with prostate cancer. Limitations include that second-line treatment after surgical or medical androgen deprivation therapy was used to define CRPC because biochemical and radiologic data that directly indicate disease progression despite androgen deprivation therapy are not available in claims data. Additionally, procedure codes in Medicare claims data for radiation therapy do not specify the anatomic target, and diagnosis codes for fractures may capture fractures due to causes other than pathologic processes (including trauma or osteoporosis). Finally, detailed information on factors that confound the relation between BTA and the incidence of SREs was not available.

## 5. Conclusions

In this large cohort of elderly men with CRPC in the US, SREs were common, with most occurring within 1 year after cohort entry. Incidence rates of SREs were slightly numerically lower following BTA use than before such use and numerically higher among patients with a history of SREs before cohort entry compared with patients who did not have such a history. The lower incidence rate after BTA use than before such use and the higher incidence rate in patients with versus without a history of SREs may be confounded by indication and other factors that cannot be adequately assessed in claims data. These findings provide a basis for future real-world studies of the incidence of SREs and the effect of BTAs and other therapeutic strategies in men with CRPC.

## Figures and Tables

**Table 1:** Demographic and clinical characteristics of the study cohort (N = 2,234).

Variable	Number of Patients (%)
Race	
White	1,867 (83.6)
Black	218 (9.8)
Asian	46 (2.1)
Hispanic	48 (2.1)
Other or unknown^a^	55 (2.5)
Age at cohort entry, years	
Mean (SD)	76.6 (6.2)
Distribution	
65-69	297 (13.3)
70-74	625 (28.0)
75-79	595 (26.6)
80-84	451 (20.2)
85+	266 (11.9)
SRE prior to cohort entry^b^	1,252 (56.0)
Use of bone-targeted agent	
First use of bone-targeted agent prior to cohort entry^c^	1,213 (54.3)
First use of bone-targeted agent after cohort entry	326 (14.6)
No use of bone-targeted agents	695 (31.1)
Length of follow-up, months	
Mean (SD)	10.6 (11.6)
Distribution	
< 6 months	960 (43.0)
6 months to 1 year	590 (26.4)
> 1 to 1.5 years	317 (14.2)
> 1.5 to 2 years	152 (6.8)
> 2 years	215 (9.6)

SD = standard deviation.

^a^ Categories were combined to avoid reporting a count of < 11.

^b^  Identified in the Medicare Provider Analysis and Review file, carrier claims, or outpatient claims anytime between the initial date of prostate cancer diagnosis and the cohort entry date.

^c^ Identified any time between the initial date of prostate cancer diagnosis and the cohort entry date.

**Table 2:** Demographic characteristics of patients with and without SREs (N = 2,234) during follow-up.

Variable	Number of Patients With SRE (%), (N = 896)	Number of Patients Without SRE (%), (N = 1,338)
Race		
White	769 (85.8)	1,098 (82.1)
Black	72 (8.0)	146 (10.9)
Asian	16 (1.8)	30 (2.2)
Hispanic	12 (1.3)	36 (2.7)
Other or unknown^a^	27 (3.0)	28 (2.1)
Age at cohort entry, years		
Mean (SD)	75.5 (5.9)	77.3 (6.3)
Distribution		
65-69	143 (16.0)	154 (11.5)
70-74	293 (32.7)	332 (24.8)
75-79	231 (25.8)	364 (27.2)
80-84	156 (17.4)	295 (22.0)
85+	73 (8.1)	193 (14.4)

^a^  Categories were combined to avoid reporting a count of < 11.

SRE = skeletal-related event.

**Table 3:** Number of first cases of SRE.

SRE	Cases (% of Total Cohort)
Radiation therapy	609 (27.3)
Fracture	266 (11.9)
Spinal cord compression	37 (1.7)
Bone surgery	22 (1.0)
Total (fracture, bone surgery, radiation therapy, or spinal cord compression)	896 (40.1)

*Notes*. Cases identified using Medicare Provider Analysis and Review file, carrier claims (Physician/Supplier Part B), and outpatient claims. SREs on the date of the initial SRE during follow-up were identified. The sum of the cases of fracture, bone surgery, radiation, and spinal cord compression is greater than the total number of SRE cases, as patients may have had more than one type of SRE on the date of the first SRE.

SRE = skeletal-related event.

**Table 4:** Incidence rates of SREs among all person-time and stratified by initial bone-targeted agent use and history of SRE before cohort entry.

Person-Time Included	Patients	Person-Months	Cases	Incidence Rate per 100 Person-Months (95% CI)
All person-time (primary analysis)	2,234	23,716	896	3.78 (3.53-4.03)
Bone-targeted agent use (secondary analysis)				
Person-time before any use	1,021	7,429	309	4.16 (3.71-4.65)
Person-time after any use	1,539	16,287	587	3.60 (3.32-3.91)
History of SRE before cohort entry (secondary analysis)				
Patients with no history of SRE	982	11,549	385	3.33 (3.01-3.68)
Patients with history of SRE	1,252	12,167	511	4.20 (3.84-4.58)

CI = confidence interval. SRE = skeletal-related event.

*Note*. The sum of patients contributing person-time before use of any bone-targeted agent and after use of any bone-targeted agent is greater than the total number of patients, as patients may have contributed person-time to both groups (see methods for details).

**Table 5:** Incidence of SREs stratified by duration of androgen deprivation therapy before cohort entry.

Duration of ADT ^a^	Cases	Person-Months	Incidence Rate per 100 Person-Months (95% CI)
0 to 1 years	240	4,947	4.85 (4.26-5.51)
>1 to 2 years	245	5,976	4.10 (3.60-4.65)
>2 to 3 years	143	4,306	3.32 (2.80-3.91)
>3 to 5 years	146	4,560	3.20 (2.70-3.77)
>5 years	122	3,928	3.11 (2.58-3.71)

CI = confidence interval; SRE = skeletal-related event; ADT = androgen deprivation therapy.

*Note*. Cases identified using Medicare Provider Analysis and Review file, Carrier claims (Physician/Supplier Part B), and Outpatient claims.

^a^Defined as the interval from date of surgical castration or starting date of medical androgen deprivation therapy to date of cohort entry.

## Data Availability

This study was conducted using data from the National Cancer Institute's (NCI's) Surveillance, Epidemiology, and End Results program in the United States and was guided by a data use agreement between NCI and RTI Health Solutions. It was reviewed by RTI International's institutional review board and received an initial exemption on February 18, 2016, for initial analyses on the cohort and a subsequent exemption for additional analyses on January 8, 2018.

## Supplementary Materials

Supplementary Materials Table S-1. ICD-9 diagnosis codes used to identify fracture in Medicare claims. Table S-2. Codes used to identify other skeletal-related events (radiation therapy, surgery on bone, and spinal cord compression) in Medicare claims.
