# Supplementary material for: Incidence of Skeletal-Related Events in Patients with Castration-Resistant Prostate Cancer: An Observational Retrospective Cohort Study in the US
Source: Prostate Cancer. 2019 Jul 9;2019:5971615. doi: 10.1155/2019/5971615 (PMC6652049; doi:10.1155/2019/5971615)
Supplement: Supplementary Materials — Table S-1. ICD-9 diagnosis codes used to identify fracture in Medicare claims. Table S-2. Codes used to identify other skeletal-related events (radiation therapy, surgery on bone, and spinal cord compression) in Medicare claims. [file 5971615.f1.docx]

# SUPPLEMENTARY TABLES

Table S-1. Diagnosis and procedure codes to identify skeletal-related events in Medicare claims

| **ICD-9 diagnosis codes to identify fracture** | |
| --- | --- |
| Code | Description |
| 7331 | Pathologic fracture |
| 73310 | Pathologic fracture, unspecified site |
| 73311 | Pathologic fracture of humerus |
| 73312 | Pathologic fracture of distal radius and ulna |
| 73313 | Pathologic fracture of vertebrae convert |
| 73314 | Pathologic fracture of neck and femur convert |
| 73315 | Pathologic fracture of other specified part of femur convert |
| 73316 | Pathologic fracture of tibia or fibula convert |
| 73319 | Pathologic fracture of other specified site |
| 73393 | Stress fracture of tibia or fibula |
| 73394 | Stress fracture of the metatarsals |
| 73395 | Stress fracture of other bone |
| 73396 | Stress fracture of femoral neck |
| 73397 | Stress fracture of shaft of femur |
| 73398 | Stress fracture of pelvis |
| 800 | Fracture of vault of skull |
| 8000 | Closed fracture of vault of skull without mention of intracranial injury |
| 80000 | Closed fracture of vault of skull without mention of intracranial injury, unspecified state of consciousness |
| 80001 | Closed fracture of vault of skull without mention of intracranial injury, with no loss of consciousness |
| 80002 | Closed fracture of vault of skull without mention of intracranial injury, with brief [less than one hour] loss of consciousness |
| 80003 | Closed fracture of vault of skull without mention of intracranial injury, with moderate [1‑24 hours] loss of consciousness |
| 80004 | Closed fracture of vault of skull without mention of intracranial injury, with prolonged [more than 24 hours] loss of consciousness and return to pre-existing conscious level |
| 80005 | Closed fracture of vault of skull without mention of intracranial injury, with prolonged [more than 24 hours] loss of consciousness, without return to pre-existing conscious level |
| 80006 | Closed fracture of vault of skull without mention of intracranial injury, with loss of consciousness of unspecified duration |
| 80009 | Closed fracture of vault of skull without mention of intracranial injury, with concussion, unspecified |
| 8001 | Closed fracture of vault of skull with cerebral laceration and contusion |
| 80010 | Closed fracture of vault of skull with cerebral laceration and contusion, unspecified state of consciousness |
| 80011 | Closed fracture of vault of skull with cerebral laceration and contusion, with no loss of consciousness |
| 80012 | Closed fracture of vault of skull with cerebral laceration and contusion, with brief [less than one hour] loss of consciousness |
| 80013 | Closed fracture of vault of skull with cerebral laceration and contusion, with moderate [1‑24 hours] loss of consciousness |
| 80014 | Closed fracture of vault of skull with cerebral laceration and contusion, with prolonged [more than 24 hours] loss of consciousness and return to pre-existing conscious level |
| 80015 | Closed fracture of vault of skull with cerebral laceration and contusion, with prolonged [more than 24 hours] loss of consciousness, without return to pre-existing conscious level |
| 80016 | Closed fracture of vault of skull with cerebral laceration and contusion, with loss of consciousness of unspecified duration |
| 80019 | Closed fracture of vault of skull with cerebral laceration and contusion, with concussion, unspecified |
| 8002 | Closed fracture of vault of skull with subarachnoid subdural and extradural hemorrhage |
| 80020 | Closed fracture of vault of skull with subarachnoid, subdural, and extradural hemorrhage, unspecified state of consciousness |
| 80021 | Closed fracture of vault of skull with subarachnoid, subdural, and extradural hemorrhage, with no loss of consciousness |
| 80022 | Closed fracture of vault of skull with subarachnoid, subdural, and extradural hemorrhage, with brief [less than one hour] loss of consciousness |
| 80023 | Closed fracture of vault of skull with subarachnoid, subdural, and extradural hemorrhage, with moderate [1-24 hours] loss of consciousness |
| 80024 | Closed fracture of vault of skull with subarachnoid, subdural, and extradural hemorrhage, with prolonged [more than 24 hours] loss of consciousness and return to pre‑existing conscious level |
| 80025 | Closed fracture of vault of skull with subarachnoid, subdural, and extradural hemorrhage, with prolonged [more than 24 hours] loss of consciousness, without return to pre-existing conscious level |
| 80026 | Closed fracture of vault of skull with subarachnoid, subdural, and extradural hemorrhage, with loss of consciousness of unspecified duration |
| 80029 | Closed fracture of vault of skull with subarachnoid, subdural, and extradural hemorrhage, with concussion, unspecified |
| 8003 | Closed fracture of vault of skull with other and unspecified intracranial hemorrhage |
| 80030 | Closed fracture of vault of skull with other and unspecified intracranial hemorrhage, unspecified state of consciousness |
| 80031 | Closed fracture of vault of skull with other and unspecified intracranial hemorrhage, with no loss of consciousness |
| 80032 | Closed fracture of vault of skull with other and unspecified intracranial hemorrhage, with brief [less than one hour] loss of consciousness |
| 80033 | Closed fracture of vault of skull with other and unspecified intracranial hemorrhage, with moderate [1-24 hours] loss of consciousness |
| 80034 | Closed fracture of vault of skull with other and unspecified intracranial hemorrhage, with prolonged [more than 24 hours] loss of consciousness and return to pre-existing conscious level |
| 80035 | Closed fracture of vault of skull with other and unspecified intracranial hemorrhage, with prolonged [more than 24 hours] loss of consciousness, without return to pre-existing conscious level |
| 80036 | Closed fracture of vault of skull with other and unspecified intracranial hemorrhage, with loss of consciousness of unspecified duration |
| 80039 | Closed fracture of vault of skull with other and unspecified intracranial hemorrhage, with concussion, unspecified |
| 8004 | Closed fracture of vault of skull with intracranial injury of other and unspecified nature |
| 80040 | Closed fracture of vault of skull with intracranial injury of other and unspecified nature, unspecified state of consciousness |
| 80041 | Closed fracture of vault of skull with intracranial injury of other and unspecified nature, with no loss of consciousness |
| 80042 | Closed fracture of vault of skull with intracranial injury of other and unspecified nature, with brief [less than one hour] loss of consciousness |
| 80043 | Closed fracture of vault of skull with intracranial injury of other and unspecified nature, with moderate [1-24 hours] loss of consciousness |
| 80044 | Closed fracture of vault of skull with intracranial injury of other and unspecified nature, with prolonged [more than 24 hours] loss of consciousness and return to pre-existing conscious level |
| 80045 | Closed fracture of vault of skull with intracranial injury of other and unspecified nature, with prolonged [more than 24 hours] loss of consciousness, without return to pre-existing conscious level |
| 80046 | Closed fracture of vault of skull with intracranial injury of other and unspecified nature, with loss of consciousness of unspecified duration |
| 80049 | Closed fracture of vault of skull with intracranial injury of other and unspecified nature, with concussion, unspecified |
| 8005 | Open fracture of vault of skull without mention of intracranial injury |
| 80050 | Open fracture of vault of skull without mention of intracranial injury, unspecified state of consciousness |
| 80051 | Open fracture of vault of skull without mention of intracranial injury, with no loss of consciousness |
| 80052 | Open fracture of vault of skull without mention of intracranial injury, with brief [less than one hour] loss of consciousness |
| 80053 | Open fracture of vault of skull without mention of intracranial injury, with moderate [1‑24 hours] loss of consciousness |
| 80054 | Open fracture of vault of skull without mention of intracranial injury, with prolonged [more than 24 hours] loss of consciousness and return to pre-existing conscious level |
| 80055 | Open fracture of vault of skull without mention of intracranial injury, with prolonged [more than 24 hours] loss of consciousness, without return to pre-existing conscious level |
| 80056 | Open fracture of vault of skull without mention of intracranial injury, with loss of consciousness of unspecified duration |
| 80059 | Open fracture of vault of skull without mention of intracranial injury, with concussion, unspecified |
| 8006 | Open fracture of vault of skull with cerebral laceration and contusion |
| 80060 | Open fracture of vault of skull with cerebral laceration and contusion, unspecified state of consciousness |
| 80061 | Open fracture of vault of skull with cerebral laceration and contusion, with no loss of consciousness |
| 80062 | Open fracture of vault of skull with cerebral laceration and contusion, with brief [less than one hour] loss of consciousness |
| 80063 | Open fracture of vault of skull with cerebral laceration and contusion, with moderate [1‑24 hours] loss of consciousness |
| 80064 | Open fracture of vault of skull with cerebral laceration and contusion, with prolonged [more than 24 hours] loss of consciousness and return to pre-existing conscious level |
| 80065 | Open fracture of vault of skull with cerebral laceration and contusion, with prolonged [more than 24 hours] loss of consciousness, without return to pre-existing conscious level |
| 80066 | Open fracture of vault of skull with cerebral laceration and contusion, with loss of consciousness of unspecified duration |
| 80069 | Open fracture of vault of skull with cerebral laceration and contusion, with concussion, unspecified |
| 8007 | Open fracture of vault of skull with subarachnoid subdural and extradural hemorrhage |
| 80070 | Open fracture of vault of skull with subarachnoid, subdural, and extradural hemorrhage, unspecified state of consciousness |
| 80071 | Open fracture of vault of skull with subarachnoid, subdural, and extradural hemorrhage, with no loss of consciousness |
| 80072 | Open fracture of vault of skull with subarachnoid, subdural, and extradural hemorrhage, with brief [less than one hour] loss of consciousness |
| 80073 | Open fracture of vault of skull with subarachnoid, subdural, and extradural hemorrhage, with moderate [1-24 hours] loss of consciousness |
| 80074 | Open fracture of vault of skull with subarachnoid, subdural, and extradural hemorrhage, with prolonged [more than 24 hours] loss of consciousness and return to pre-existing conscious level |
| 80075 | Open fracture of vault of skull with subarachnoid, subdural, and extradural hemorrhage, with prolonged [more than 24 hours] loss of consciousness, without return to pre-existing conscious level |
| 80076 | Open fracture of vault of skull with subarachnoid, subdural, and extradural hemorrhage, with loss of consciousness of unspecified duration |
| 80079 | Open fracture of vault of skull with subarachnoid, subdural, and extradural hemorrhage, with concussion, unspecified |
| 8008 | Open fracture of vault of skull with other and unspecified intracranial hemorrhage |
| 80080 | Open fracture of vault of skull with other and unspecified intracranial hemorrhage, unspecified state of consciousness |
| 80081 | Open fracture of vault of skull with other and unspecified intracranial hemorrhage, with no loss of consciousness |
| 80082 | Open fracture of vault of skull with other and unspecified intracranial hemorrhage, with brief [less than one hour] loss of consciousness |
| 80083 | Open fracture of vault of skull with other and unspecified intracranial hemorrhage, with moderate [1-24 hours] loss of consciousness |
| 80084 | Open fracture of vault of skull with other and unspecified intracranial hemorrhage, with prolonged [more than 24 hours] loss of consciousness and return to pre-existing conscious level |
| 80085 | Open fracture of vault of skull with other and unspecified intracranial hemorrhage, with prolonged [more than 24 hours] loss of consciousness, without return to pre-existing conscious level |
| 80086 | Open fracture of vault of skull with other and unspecified intracranial hemorrhage, with loss of consciousness of unspecified duration |
| 80089 | Open fracture of vault of skull with other and unspecified intracranial hemorrhage, with concussion, unspecified |
| 8009 | Open fracture of vault of skull with intracranial injury of other and unspecified nature |
| 80090 | Open fracture of vault of skull with intracranial injury of other and unspecified nature, unspecified state of consciousness |
| 80091 | Open fracture of vault of skull with intracranial injury of other and unspecified nature, with no loss of consciousness |
| 80092 | Open fracture of vault of skull with intracranial injury of other and unspecified nature, with brief [less than one hour] loss of consciousness |
| 80093 | Open fracture of vault of skull with intracranial injury of other and unspecified nature, with moderate [1-24 hours] loss of consciousness |
| 80094 | Open fracture of vault of skull with intracranial injury of other and unspecified nature, with prolonged [more than 24 hours] loss of consciousness and return to pre-existing conscious level |
| 80095 | Open fracture of vault of skull with intracranial injury of other and unspecified nature, with prolonged [more than 24 hours] loss of consciousness, without return to pre-existing conscious level |
| 80096 | Open fracture of vault of skull with intracranial injury of other and unspecified nature, with loss of consciousness of unspecified duration |
| 80099 | Open fracture of vault of skull with intracranial injury of other and unspecified nature, with concussion, unspecified |
| 801 | Fracture of base of skull |
| 8010 | Closed fracture of base of skull without mention of intracranial injury |
| 80100 | Closed fracture of base of skull without mention of intracranial injury, unspecified state of consciousness |
| 80101 | Closed fracture of base of skull without mention of intracranial injury, with no loss of consciousness |
| 80102 | Closed fracture of base of skull without mention of intracranial injury, with brief [less than one hour] loss of consciousness |
| 80103 | Closed fracture of base of skull without mention of intracranial injury, with moderate [1‑24 hours] loss of consciousness |
| 80104 | Closed fracture of base of skull without mention of intracranial injury, with prolonged [more than 24 hours] loss of consciousness and return to pre-existing conscious level |
| 80105 | Closed fracture of base of skull without mention of intracranial injury, with prolonged [more than 24 hours] loss of consciousness, without return to pre-existing conscious level |
| 80106 | Closed fracture of base of skull without mention of intracranial injury, with loss of consciousness of unspecified duration |
| 80109 | Closed fracture of base of skull without mention of intracranial injury, with concussion, unspecified |
| 8011 | Closed fracture of base of skull with cerebral laceration and contusion |
| 80110 | Closed fracture of base of skull with cerebral laceration and contusion, unspecified state of consciousness |
| 80111 | Closed fracture of base of skull with cerebral laceration and contusion, with no loss of consciousness |
| 80112 | Closed fracture of base of skull with cerebral laceration and contusion, with brief [less than one hour] loss of consciousness |
| 80113 | Closed fracture of base of skull with cerebral laceration and contusion, with moderate [1‑24 hours] loss of consciousness |
| 80114 | Closed fracture of base of skull with cerebral laceration and contusion, with prolonged [more than 24 hours] loss of consciousness and return to pre-existing conscious level |
| 80115 | Closed fracture of base of skull with cerebral laceration and contusion, with prolonged [more than 24 hours] loss of consciousness, without return to pre-existing conscious level |
| 80116 | Closed fracture of base of skull with cerebral laceration and contusion, with loss of consciousness of unspecified duration |
| 80119 | Closed fracture of base of skull with cerebral laceration and contusion, with concussion, unspecified |
| 802 | Fracture of face bones |
| 8020 | Closed fracture of nasal bones |
| 8021 | Open fracture of nasal bones |
| 8022 | Closed fracture of mandible |
| 80220 | Closed fracture of mandible, unspecified site |
| 80221 | Closed fracture of mandible, condylar process |
| 80222 | Closed fracture of mandible, subcondylar |
| 80223 | Closed fracture of mandible, coronoid process |
| 80224 | Closed fracture of mandible, ramus, unspecified |
| 80225 | Closed fracture of mandible, angle of jaw |
| 80226 | Closed fracture of mandible, symphysis of body |
| 80227 | Closed fracture of mandible, alveolar border of body |
| 80228 | Closed fracture of mandible, body, other and unspecified |
| 80229 | Closed fracture of mandible, multiple sites |
| 8023 | Open fracture of mandible |
| 80230 | Open fracture of mandible, unspecified site |
| 80231 | Open fracture of mandible, condylar process |
| 80232 | Open fracture of mandible, subcondylar |
| 80233 | Open fracture of mandible, coronoid process |
| 80234 | Open fracture of mandible, ramus, unspecified |
| 80235 | Open fracture of mandible, angle of jaw |
| 80236 | Open fracture of mandible, symphysis of body |
| 80237 | Open fracture of mandible, alveolar border of body |
| 80238 | Open fracture of mandible, body, other and unspecified |
| 80239 | Open fracture of mandible, multiple sites |
| 8024 | Closed fracture of malar and maxillary bones |
| 8025 | Open fracture of malar and maxillary bones |
| 8026 | Closed fracture of orbital floor (blow-out) |
| 8027 | Open fracture of orbital floor (blow-out) |
| 8028 | Closed fracture of other facial bones |
| 8029 | Open fracture of other facial bones |
| 803 | Other and unqualified skull fractures |
| 8030 | Other closed skull fracture without mention of intracranial injury |
| 80300 | Other closed skull fracture without mention of intracranial injury, unspecified state of consciousness |
| 80301 | Other closed skull fracture without mention of intracranial injury, with no loss of consciousness |
| 80302 | Other closed skull fracture without mention of intracranial injury, with brief [less than one hour] loss of consciousness |
| 80303 | Other closed skull fracture without mention of intracranial injury, with moderate [1-24 hours] loss of consciousness |
| 80304 | Other closed skull fracture without mention of intracranial injury, with prolonged [more than 24 hours] loss of consciousness and return to pre-existing conscious level |
| 80305 | Other closed skull fracture without mention of intracranial injury, with prolonged [more than 24 hours] loss of consciousness, without return to pre-existing conscious level |
| 80306 | Other closed skull fracture without mention of intracranial injury, with loss of consciousness of unspecified duration |
| 80309 | Other closed skull fracture without mention of intracranial injury, with concussion, unspecified |
| 8031 | Other closed skull fracture with cerebral laceration and contusion |
| 80310 | Other closed skull fracture with cerebral laceration and contusion, unspecified state of consciousness |
| 80311 | Other closed skull fracture with cerebral laceration and contusion, with no loss of consciousness |
| 80312 | Other closed skull fracture with cerebral laceration and contusion, with brief [less than one hour] loss of consciousness |
| 80313 | Other closed skull fracture with cerebral laceration and contusion, with moderate [1‑24 hours] loss of consciousness |
| 80314 | Other closed skull fracture with cerebral laceration and contusion, with prolonged [more than 24 hours] loss of consciousness and return to pre-existing conscious level |
| 80315 | Other closed skull fracture with cerebral laceration and contusion, with prolonged [more than 24 hours] loss of consciousness, without return to pre-existing conscious level |
| 80316 | Other closed skull fracture with cerebral laceration and contusion, with loss of consciousness of unspecified duration |
| 80319 | Other closed skull fracture with cerebral laceration and contusion, with concussion, unspecified |
| 8032 | Other closed skull fracture with subarachnoid subdural and extradural hemorrhage |
| 80320 | Other closed skull fracture with subarachnoid, subdural, and extradural hemorrhage, unspecified state of consciousness |
| 80321 | Other closed skull fracture with subarachnoid, subdural, and extradural hemorrhage, with no loss of consciousness |
| 80322 | Other closed skull fracture with subarachnoid, subdural, and extradural hemorrhage, with brief [less than one hour] loss of consciousness |
| 80323 | Other closed skull fracture with subarachnoid, subdural, and extradural hemorrhage, with moderate [1-24 hours] loss of consciousness |
| 80324 | Other closed skull fracture with subarachnoid, subdural, and extradural hemorrhage, with prolonged [more than 24 hours] loss of consciousness and return to pre-existing conscious level |
| 80325 | Other closed skull fracture with subarachnoid, subdural, and extradural hemorrhage, with prolonged [more than 24 hours] loss of consciousness, without return to pre-existing conscious level |
| 80326 | Other closed skull fracture with subarachnoid, subdural, and extradural hemorrhage, with loss of consciousness of unspecified duration |
| 80329 | Other closed skull fracture with subarachnoid, subdural, and extradural hemorrhage, with concussion, unspecified |
| 8033 | Closed skull fracture with other and unspecified intracranial hemorrhage |
| 80330 | Other closed skull fracture with other and unspecified intracranial hemorrhage, unspecified state of unconsciousness |
| 80331 | Other closed skull fracture with other and unspecified intracranial hemorrhage, with no loss of consciousness |
| 80332 | Other closed skull fracture with other and unspecified intracranial hemorrhage, with brief [less than one hour] loss of consciousness |
| 80333 | Other closed skull fracture with other and unspecified intracranial hemorrhage, with moderate [1-24 hours] loss of consciousness |
| 80334 | Other closed skull fracture with other and unspecified intracranial hemorrhage, with prolonged [more than 24 hours] loss of consciousness and return to pre-existing conscious level |
| 80335 | Other closed skull fracture with other and unspecified intracranial hemorrhage, with prolonged [more than 24 hours] loss of consciousness, without return to pre-existing conscious level |
| 80336 | Other closed skull fracture with other and unspecified intracranial hemorrhage, with loss of consciousness of unspecified duration |
| 80339 | Other closed skull fracture with other and unspecified intracranial hemorrhage, with concussion, unspecified |
| 8034 | Closed skull fracture with intracranial injury of other and unspecified nature |
| 80340 | Other closed skull fracture with intracranial injury of other and unspecified nature, unspecified state of consciousness |
| 80341 | Other closed skull fracture with intracranial injury of other and unspecified nature, with no loss of consciousness |
| 80342 | Other closed skull fracture with intracranial injury of other and unspecified nature, with brief [less than one hour] loss of consciousness |
| 80343 | Other closed skull fracture with intracranial injury of other and unspecified nature, with moderate [1-24 hours] loss of consciousness |
| 80344 | Other closed skull fracture with intracranial injury of other and unspecified nature, with prolonged [more than 24 hours] loss of consciousness and return to pre-existing conscious level |
| 80345 | Other closed skull fracture with intracranial injury of other and unspecified nature, with prolonged [more than 24 hours] loss of consciousness, without return to pre-existing conscious level |
| 80346 | Other closed skull fracture with intracranial injury of other and unspecified nature, with loss of consciousness of unspecified duration |
| 80349 | Other closed skull fracture with intracranial injury of other and unspecified nature, with concussion, unspecified |
| 8035 | Other open skull fracture without mention of intracranial injury |
| 80350 | Other open skull fracture without mention of injury, unspecified state of consciousness |
| 80351 | Other open skull fracture without mention of intracranial injury, with no loss of consciousness |
| 80352 | Other open skull fracture without mention of intracranial injury, with brief [less than one hour] loss of consciousness |
| 80353 | Other open skull fracture without mention of intracranial injury, with moderate [1-24 hours] loss of consciousness |
| 80354 | Other open skull fracture without mention of intracranial injury, with prolonged [more than 24 hours] loss of consciousness and return to pre-existing conscious level |
| 80355 | Other open skull fracture without mention of intracranial injury, with prolonged [more than 24 hours] loss of consciousness, without return to pre-existing conscious level |
| 80356 | Other open skull fracture without mention of intracranial injury, with loss of consciousness of unspecified duration |
| 80359 | Other open skull fracture without mention of intracranial injury, with concussion, unspecified |
| 8036 | Other open skull fracture with cerebral laceration and contusion |
| 80360 | Other open skull fracture with cerebral laceration and contusion, unspecified state of consciousness |
| 80361 | Other open skull fracture with cerebral laceration and contusion, with no loss of consciousness |
| 80362 | Other open skull fracture with cerebral laceration and contusion, with brief [less than one hour] loss of consciousness |
| 80363 | Other open skull fracture with cerebral laceration and contusion, with moderate [1‑24 hours] loss of consciousness |
| 80364 | Other open skull fracture with cerebral laceration and contusion, with prolonged [more than 24 hours] loss of consciousness and return to pre-existing conscious level |
| 80365 | Other open skull fracture with cerebral laceration and contusion, with prolonged [more than 24 hours] loss of consciousness, without return to pre-existing conscious level |
| 80366 | Other open skull fracture with cerebral laceration and contusion, with loss of consciousness of unspecified duration |
| 80369 | Other open skull fracture with cerebral laceration and contusion, with concussion, unspecified |
| 8037 | Other open skull fracture with subarachnoid subdural and extradural hemorrhage |
| 80370 | Other open skull fracture with subarachnoid, subdural, and extradural hemorrhage, unspecified state of consciousness |
| 80371 | Other open skull fracture with subarachnoid, subdural, and extradural hemorrhage, with no loss of consciousness |
| 80372 | Other open skull fracture with subarachnoid, subdural, and extradural hemorrhage, with brief [less than one hour] loss of consciousness |
| 80373 | Other open skull fracture with subarachnoid, subdural, and extradural hemorrhage, with moderate [1-24 hours] loss of consciousness |
| 80374 | Other open skull fracture with subarachnoid, subdural, and extradural hemorrhage, with prolonged [more than 24 hours] loss of consciousness and return to pre-existing conscious level |
| 80375 | Other open skull fracture with subarachnoid, subdural, and extradural hemorrhage, with prolonged [more than 24 hours] loss of consciousness, without return to pre-existing conscious level |
| 80376 | Other open skull fracture with subarachnoid, subdural, and extradural hemorrhage, with loss of consciousness of unspecified duration |
| 80379 | Other open skull fracture with subarachnoid, subdural, and extradural hemorrhage, with concussion, unspecified |
| 8038 | Other open skull fracture with other and unspecified intracranial hemorrhage |
| 80380 | Other open skull fracture with other and unspecified intracranial hemorrhage, unspecified state of consciousness |
| 80381 | Other open skull fracture with other and unspecified intracranial hemorrhage, with no loss of consciousness |
| 80382 | Other open skull fracture with other and unspecified intracranial hemorrhage, with brief [less than one hour] loss of consciousness |
| 80383 | Other open skull fracture with other and unspecified intracranial hemorrhage, with moderate [1-24 hours] loss of consciousness |
| 80384 | Other open skull fracture with other and unspecified intracranial hemorrhage, with prolonged [more than 24 hours] loss of consciousness and return to pre-existing conscious level |
| 80385 | Other open skull fracture with other and unspecified intracranial hemorrhage, with prolonged [more than 24 hours] loss of consciousness, without return to pre-existing conscious level |
| 80386 | Other open skull fracture with other and unspecified intracranial hemorrhage, with loss of consciousness of unspecified duration |
| 80389 | Other open skull fracture with other and unspecified intracranial hemorrhage, with concussion, unspecified |
| 8039 | Other open skull fracture with intracranial injury of other and unspecified nature |
| 80390 | Other open skull fracture with intracranial injury of other and unspecified nature, unspecified state of consciousness |
| 80391 | Other open skull fracture with intracranial injury of other and unspecified nature, with no loss of consciousness |
| 80392 | Other open skull fracture with intracranial injury of other and unspecified nature, with brief [less than one hour] loss of consciousness |
| 80393 | Other open skull fracture with intracranial injury of other and unspecified nature, with moderate [1-24 hours] loss of consciousness |
| 80394 | Other open skull fracture with intracranial injury of other and unspecified nature, with prolonged [more than 24 hours] loss of consciousness and return to pre-existing conscious level |
| 80395 | Other open skull fracture with intracranial injury of other and unspecified nature, with prolonged [more than 24 hours] loss of consciousness, without return to pre-existing conscious level |
| 80396 | Other open skull fracture with intracranial injury of other and unspecified nature, with loss of consciousness of unspecified duration |
| 80399 | Other open skull fracture with intracranial injury of other and unspecified nature, with concussion, unspecified |
| 804 | Multiple fractures involving skull or face with other bones |
| 8040 | Closed fractures involving skull or face with other bones without mention of intracranial injury |
| 80400 | Closed fractures involving skull or face with other bones, without mention of intracranial injury, unspecified state of consciousness |
| 80401 | Closed fractures involving skull or face with other bones, without mention of intracranial injury, with no loss of consciousness |
| 80402 | Closed fractures involving skull or face with other bones, without mention of intracranial injury, with brief [less than one hour] loss of consciousness |
| 80403 | Closed fractures involving skull or face with other bones, without mention of intracranial injury, with moderate [1-24 hours] loss of consciousness |
| 80404 | Closed fractures involving skull or face with other bones, without mention or intracranial injury, with prolonged [more than 24 hours] loss of consciousness and return to pre-existing conscious level |
| 80405 | Closed fractures involving skull of face with other bones, without mention of intracranial injury, with prolonged [more than 24 hours] loss of consciousness, without return to pre‑existing conscious level |
| 80406 | Closed fractures involving skull of face with other bones, without mention of intracranial injury, with loss of consciousness of unspecified duration |
| 80409 | Closed fractures involving skull of face with other bones, without mention of intracranial injury, with concussion, unspecified |
| 8041 | Closed fractures involving skull or face with other bones with cerebral laceration and contusion |
| 80410 | Closed fractures involving skull or face with other bones, with cerebral laceration and contusion, unspecified state of consciousness |
| 80411 | Closed fractures involving skull or face with other bones, with cerebral laceration and contusion, with no loss of consciousness |
| 80412 | Closed fractures involving skull or face with other bones, with cerebral laceration and contusion, with brief [less than one hour] loss of consciousness |
| 80413 | Closed fractures involving skull or face with other bones, with cerebral laceration and contusion, with moderate [1-24 hours] loss of consciousness |
| 80414 | Closed fractures involving skull or face with other bones, with cerebral laceration and contusion, with prolonged [more than 24 hours] loss of consciousness and return to pre‑existing conscious level |
| 80415 | Closed fractures involving skull or face with other bones, with cerebral laceration and contusion, with prolonged [more than 24 hours] loss of consciousness, without return to pre-existing conscious level |
| 80416 | Closed fractures involving skull or face with other bones, with cerebral laceration and contusion, with loss of consciousness of unspecified duration |
| 80419 | Closed fractures involving skull or face with other bones, with cerebral laceration and contusion, with concussion, unspecified |
| 8042 | Closed fractures involving skull or face with other bones with subarachnoid subdural and extradural hemorrhage |
| 80420 | Closed fractures involving skull or face with other bones with subarachnoid, subdural, and extradural hemorrhage, unspecified state of consciousness |
| 80421 | Closed fractures involving skull or face with other bones with subarachnoid, subdural, and extradural hemorrhage, with no loss of consciousness |
| 80422 | Closed fractures involving skull or face with other bones with subarachnoid, subdural, and extradural hemorrhage, with brief [less than one hour] loss of consciousness |
| 80423 | Closed fractures involving skull or face with other bones with subarachnoid, subdural, and extradural hemorrhage, with moderate [1-24 hours] loss of consciousness |
| 80424 | Closed fractures involving skull or face with other bones with subarachnoid, subdural, and extradural hemorrhage, with prolonged [more than 24 hours] loss of consciousness and return to pre-existing conscious level |
| 80425 | Closed fractures involving skull or face with other bones with subarachnoid, subdural, and extradural hemorrhage, with prolonged [more than 24 hours] loss of consciousness, without return to pre-existing conscious level |
| 80426 | Closed fractures involving skull or face with other bones with subarachnoid, subdural, and extradural hemorrhage, with loss of consciousness of unspecified duration |
| 80429 | Closed fractures involving skull or face with other bones with subarachnoid, subdural, and extradural hemorrhage, with concussion, unspecified |
| 8043 | Closed fractures involving skull or face with other bones, with other and unspecified intracranial hemorrhage |
| 80430 | Closed fractures involving skull or face with other bones, with other and unspecified intracranial hemorrhage, unspecified state of consciousness |
| 80431 | Closed fractures involving skull or face with other bones, with other and unspecified intracranial hemorrhage, with no loss of consciousness |
| 80432 | Closed fractures involving skull or face with other bones, with other and unspecified intracranial hemorrhage, with brief [less than one hour] loss of consciousness |
| 80433 | Closed fractures involving skull or face with other bones, with other and unspecified intracranial hemorrhage, with moderate [1-24 hours] loss of consciousness |
| 80434 | Closed fractures involving skull or face with other bones, with other and unspecified intracranial hemorrhage, with prolonged [more than 24 hours] loss of consciousness and return to pre- existing conscious level |
| 80435 | Closed fractures involving skull or face with other bones, with other and unspecified intracranial hemorrhage, with prolonged [more than 24 hours] loss of consciousness, without return to pre-existing conscious level |
| 80436 | Closed fractures involving skull or face with other bones, with other and unspecified intracranial hemorrhage, with loss of consciousness of unspecified duration |
| 80439 | Closed fractures involving skull or face with other bones, with other and unspecified intracranial hemorrhage, with concussion, unspecified |
| 8044 | Closed fractures involving skull or face with other bones with intracranial injury of other and unspecified nature |
| 80440 | Closed fractures involving skull or face with other bones, with intracranial injury of other and unspecified nature, unspecified state of consciousness |
| 80441 | Closed fractures involving skull or face with other bones, with intracranial injury of other and unspecified nature, with no loss of consciousness |
| 80442 | Closed fractures involving skull or face with other bones, with intracranial injury of other and unspecified nature, with brief [less than one hour] loss of consciousness |
| 80443 | Closed fractures involving skull or face with other bones, with intracranial injury of other and unspecified nature, with moderate [1-24 hours] loss of consciousness |
| 80444 | Closed fractures involving skull or face with other bones, with intracranial injury of other and unspecified nature, with prolonged [more than 24 hours] loss of consciousness and return to pre-existing conscious level |
| 80445 | Closed fractures involving skull or face with other bones, with intracranial injury of other and unspecified nature, with prolonged [more than 24 hours] loss of consciousness, without return to pre-existing conscious level |
| 80446 | Closed fractures involving skull or face with other bones, with intracranial injury of other and unspecified nature, with loss of consciousness of unspecified duration |
| 80449 | Closed fractures involving skull or face with other bones, with intracranial injury of other and unspecified nature, with concussion, unspecified |
| 8045 | Open fractures involving skull or face with other bones without mention of intracranial injury |
| 80450 | Open fractures involving skull or face with other bones, without mention of intracranial injury, unspecified state of consciousness |
| 80451 | Open fractures involving skull or face with other bones, without mention of intracranial injury, with no loss of consciousness |
| 80452 | Open fractures involving skull or face with other bones, without mention of intracranial injury, with brief [less than one hour] loss of consciousness |
| 80453 | Open fractures involving skull or face with other bones, without mention of intracranial injury, with moderate [1-24 hours] loss of consciousness |
| 80454 | Open fractures involving skull or face with other bones, without mention of intracranial injury, with prolonged [more than 24 hours] loss of consciousness and return to pre-existing conscious level |
| 80455 | Open fractures involving skull or face with other bones, without mention of intracranial injury, with prolonged [more than 24 hours] loss of consciousness, without return to pre‑existing conscious level |
| 80456 | Open fractures involving skull or face with other bones, without mention of intracranial injury, with loss of consciousness of unspecified duration |
| 80459 | Open fractures involving skull or face with other bones, without mention of intracranial injury, with concussion, unspecified |
| 8046 | Open fractures involving skull or face with other bones with cerebral laceration and contusion |
| 80460 | Open fractures involving skull or face with other bones, with cerebral laceration and contusion, unspecified state of consciousness |
| 80461 | Open fractures involving skull or face with other bones, with cerebral laceration and contusion, with no loss of consciousness |
| 80462 | Open fractures involving skull or face with other bones, with cerebral laceration and contusion, with brief [less than one hour] loss of consciousness |
| 80463 | Open fractures involving skull or face with other bones, with cerebral laceration and contusion, with moderate [1-24 hours] loss of consciousness |
| 80464 | Open fractures involving skull or face with other bones, with cerebral laceration and contusion, with prolonged [more than 24 hours] loss of consciousness and return to pre‑existing conscious level |
| 80465 | Open fractures involving skull or face with other bones, with cerebral laceration and contusion, with prolonged [more than 24 hours] loss of consciousness, without return to pre-existing conscious level |
| 80466 | Open fractures involving skull or face with other bones, with cerebral laceration and contusion, with loss of consciousness of unspecified duration |
| 80469 | Open fractures involving skull or face with other bones, with cerebral laceration and contusion, with concussion, unspecified |
| 8047 | Open fractures involving skull or face with other bones with subarachnoid subdural and extradural hemorrhage |
| 80470 | Open fractures involving skull or face with other bones with subarachnoid, subdural, and extradural hemorrhage, unspecified state of consciousness |
| 80471 | Open fractures involving skull or face with other bones with subarachnoid, subdural, and extradural hemorrhage, with no loss of consciousness |
| 80472 | Open fractures involving skull or face with other bones with subarachnoid, subdural, and extradural hemorrhage, with brief [less than one hour] loss of consciousness |
| 80473 | Open fractures involving skull or face with other bones with subarachnoid, subdural, and extradural hemorrhage, with moderate [1-24 hours] loss of consciousness |
| 80474 | Open fractures involving skull or face with other bones with subarachnoid, subdural, and extradural hemorrhage, with prolonged [more than 24 hours] loss of consciousness and return to pre-existing conscious level |
| 80475 | Open fractures involving skull or face with other bones with subarachnoid, subdural, and extradural hemorrhage, with prolonged [more than 24 hours] loss of consciousness, without return to pre-existing conscious level |
| 80476 | Open fractures involving skull or face with other bones with subarachnoid, subdural, and extradural hemorrhage, with loss of consciousness of unspecified duration |
| 80479 | Open fractures involving skull or face with other bones with subarachnoid, subdural, and extradural hemorrhage, with concussion, unspecified |
| 8048 | Open fractures involving skull or face with other bones with other and unspecified intracranial hemorrhage |
| 80480 | Open fractures involving skull or face with other bones, with other and unspecified intracranial hemorrhage, unspecified state of consciousness |
| 80481 | Open fractures involving skull or face with other bones, with other and unspecified intracranial hemorrhage, with no loss of consciousness |
| 80482 | Open fractures involving skull or face with other bones, with other and unspecified intracranial hemorrhage, with brief [less than one hour] loss of consciousness |
| 80483 | Open fractures involving skull or face with other bones, with other and unspecified intracranial hemorrhage, with moderate [1-24 hours] loss of consciousness |
| 80484 | Open fractures involving skull or face with other bones, with other and unspecified intracranial hemorrhage, with prolonged [more than 24 hours] loss of consciousness and return to pre-existing conscious level |
| 80485 | Open fractures involving skull or face with other bones, with other and unspecified intracranial hemorrhage, with prolonged [more than 24 hours] loss consciousness, without return to pre-existing conscious level |
| 80486 | Open fractures involving skull or face with other bones, with other and unspecified intracranial hemorrhage, with loss of consciousness of unspecified duration |
| 80489 | Open fractures involving skull or face with other bones, with other and unspecified intracranial hemorrhage, with concussion, unspecified |
| 8049 | Open fractures involving skull or face with other bones with intracranial injury of other and unspecified nature |
| 80490 | Open fractures involving skull or face with other bones, with intracranial injury of other and unspecified nature, unspecified state of consciousness |
| 80491 | Open fractures involving skull or face with other bones, with intracranial injury of other and unspecified nature, with no loss of consciousness |
| 80492 | Open fractures involving skull or face with other bones, with intracranial injury of other and unspecified nature, with brief [less than one hour] loss of consciousness |
| 80493 | Open fractures involving skull or face with other bones, with intracranial injury of other and unspecified nature, with moderate [1-24 hours] loss of consciousness |
| 80494 | Open fractures involving skull or face with other bones, with intracranial injury of other and unspecified nature, with prolonged [more than 24 hours] loss of consciousness and return to pre-existing conscious level |
| 80495 | Open fractures involving skull or face with other bones, with intracranial injury of other and unspecified nature, with prolonged [more than 24 hours] loss of consciousness without return to pre-existing conscious level |
| 80496 | Open fractures involving skull or face with other bones, with intracranial injury of other and unspecified nature, with loss of consciousness of unspecified duration |
| 80499 | Open fractures involving skull or face with other bones, with intracranial injury of other and unspecified nature, with concussion, unspecified |
| 805 | Fracture of vertebral column without mention of spinal cord injury |
| 8050 | Closed fracture of cervical vertebra without mention of spinal cord injury |
| 80500 | Closed fracture of cervical vertebra, unspecified level |
| 80501 | Closed fracture of first cervical vertebra |
| 80502 | Closed fracture of second cervical vertebra |
| 80503 | Closed fracture of third cervical vertebra |
| 80504 | Closed fracture of fourth cervical vertebra |
| 80505 | Closed fracture of fifth cervical vertebra |
| 80506 | Closed fracture of sixth cervical vertebra |
| 80507 | Closed fracture of seventh cervical vertebra |
| 80508 | Closed fracture of multiple cervical vertebrae |
| 8051 | Open fracture of cervical vertebra without mention of spinal cord injury |
| 80510 | Open fracture of cervical vertebra, unspecified level |
| 80511 | Open fracture of first cervical vertebra |
| 80512 | Open fracture of second cervical vertebra |
| 80513 | Open fracture of third cervical vertebra |
| 80514 | Open fracture of fourth cervical vertebra |
| 80515 | Open fracture of fifth cervical vertebra |
| 80516 | Open fracture of sixth cervical vertebra |
| 80517 | Open fracture of seventh cervical vertebra |
| 80518 | Open fracture of multiple cervical vertebrae |
| 8052 | Closed fracture of dorsal [thoracic] vertebra without mention of spinal cord injury |
| 8053 | Open fracture of dorsal [thoracic] vertebra without mention of spinal cord injury |
| 8054 | Closed fracture of lumbar vertebra without mention of spinal cord injury |
| 8055 | Open fracture of lumbar vertebra without mention of spinal cord injury |
| 8056 | Closed fracture of sacrum and coccyx without mention of spinal cord injury |
| 8057 | Open fracture of sacrum and coccyx without mention of spinal cord injury |
| 8058 | Closed fracture of unspecified vertebral column without mention of spinal cord injury |
| 8059 | Open fracture of unspecified vertebral column without mention of spinal cord injury |
| 806 | Fracture of vertebral column with spinal cord injury |
| 8060 | Closed fracture of cervical vertebra with spinal cord injury |
| 80600 | Closed fracture of C1-C4 level with unspecified spinal cord injury |
| 80601 | Closed fracture of C1-C4 level with complete lesion of cord |
| 80602 | Closed fracture of C1-C4 level with anterior cord syndrome |
| 80603 | Closed fracture of C1-C4 level with central cord syndrome |
| 80604 | Closed fracture of C1-C4 level with other specified spinal cord injury |
| 80605 | Closed fracture of C5-C7 level with unspecified spinal cord injury |
| 80606 | Closed fracture of C5-C7 level with complete lesion of cord |
| 80607 | Closed fracture of C5-C7 level with anterior cord syndrome |
| 80608 | Closed fracture of C5-C7 level with central cord syndrome |
| 80609 | Closed fracture of C5-C7 level with other specified spinal cord injury |
| 8061 | Open fracture of cervical vertebra with spinal cord injury |
| 80610 | Open fracture of C1-C4 level with unspecified spinal cord injury |
| 80611 | Open fracture of C1-C4 level with complete lesion of cord |
| 80612 | Open fracture of C1-C4 level with anterior cord syndrome |
| 80613 | Open fracture of C1-C4 level with central cord syndrome |
| 80614 | Open fracture of C1-C4 level with other specified spinal cord injury |
| 80615 | Open fracture of C5-C7 level with unspecified spinal cord injury |
| 80616 | Open fracture of C5-C7 level with complete lesion of cord |
| 80617 | Open fracture of C5-C7 level with anterior cord syndrome |
| 80618 | Open fracture of C5-C7 level with central cord syndrome |
| 80619 | Open fracture of C5-C7 level with other specified spinal cord injury |
| 8062 | Closed fracture of dorsal vertebra with spinal cord injury |
| 80620 | Closed fracture of T1-T6 level with unspecified spinal cord injury |
| 80621 | Closed fracture of T1-T6 level with complete lesion of cord |
| 80622 | Closed fracture of T1-T6 level with anterior cord syndrome |
| 80623 | Closed fracture of T1-T6 level with central cord syndrome |
| 80624 | Closed fracture of T1-T6 level with other specified spinal cord injury |
| 80625 | Closed fracture of T7-T12 level with unspecified spinal cord injury |
| 80626 | Closed fracture of T7-T12 level with complete lesion of cord |
| 80627 | Closed fracture of T7-T12 level with anterior cord syndrome |
| 80628 | Closed fracture of T7-T12 level with central cord syndrome |
| 80629 | Closed fracture of T7-T12 level with other specified spinal cord injury |
| 8063 | Open fracture of dorsal vertebra with spinal cord injury |
| 80630 | Open fracture of T1-T6 level with unspecified spinal cord injury |
| 80631 | Open fracture of T1-T6 level with complete lesion of cord |
| 80632 | Open fracture of T1-T6 level with anterior cord syndrome |
| 80633 | Open fracture of T1-T6 level with central cord syndrome |
| 80634 | Open fracture of T1-T6 level with other specified spinal cord injury |
| 80635 | Open fracture of T7-T12 level with unspecified spinal cord injury |
| 80636 | Open fracture of T7-T12 level with complete lesion of cord |
| 80637 | Open fracture of T7-T12 level with anterior cord syndrome |
| 80638 | Open fracture of T7-T12 level with central cord syndrome |
| 80639 | Open fracture of T7-T12 level with other specified spinal cord injury |
| 8064 | Closed fracture of lumbar spine with spinal cord injury |
| 8065 | Open fracture of lumbar spine with spinal cord injury |
| 8066 | Closed fracture of sacrum and coccyx with spinal cord injury |
| 80660 | Closed fracture of sacrum and coccyx with unspecified spinal cord injury |
| 80661 | Closed fracture of sacrum and coccyx with complete cauda equina lesion |
| 80662 | Closed fracture of sacrum and coccyx with other cauda equina injury |
| 80669 | Closed fracture of sacrum and coccyx with other spinal cord injury |
| 8067 | Open fracture of sacrum and coccyx with spinal cord injury |
| 80670 | Open fracture of sacrum and coccyx with unspecified spinal cord injury |
| 80671 | Open fracture of sacrum and coccyx with complete cauda equina lesion |
| 80672 | Open fracture of sacrum and coccyx with other cauda equina injury |
| 80679 | Open fracture of sacrum and coccyx with other spinal cord injury |
| 8068 | Closed fracture of unspecified vertebral column with spinal cord injury |
| 8069 | Open fracture of unspecified vertebral column with spinal cord injury |
| 807 | Fracture of rib(s) sternum larynx and trachea |
| 8070 | Closed fracture of rib(s) |
| 80700 | Closed fracture of rib(s), unspecified |
| 80701 | Closed fracture of one rib |
| 80702 | Closed fracture of two ribs |
| 80703 | Closed fracture of three ribs |
| 80704 | Closed fracture of four ribs |
| 80705 | Closed fracture of five ribs |
| 80706 | Closed fracture of six ribs |
| 80707 | Closed fracture of seven ribs |
| 80708 | Closed fracture of eight or more ribs |
| 80709 | Closed fracture of multiple ribs, unspecified |
| 8071 | Open fracture of rib(s) |
| 80710 | Open fracture of rib(s), unspecified |
| 80711 | Open fracture of one rib |
| 80712 | Open fracture of two ribs |
| 80713 | Open fracture of three ribs |
| 80714 | Open fracture of four ribs |
| 80715 | Open fracture of five ribs |
| 80716 | Open fracture of six ribs |
| 80717 | Open fracture of seven ribs |
| 80718 | Open fracture of eight or more ribs |
| 80719 | Open fracture of multiple ribs, unspecified |
| 8072 | Closed fracture of sternum |
| 8073 | Open fracture of sternum |
| 8074 | Flail chest |
| 808 | Fracture of pelvis |
| 8080 | Closed fracture of acetabulum |
| 8081 | Open fracture of acetabulum |
| 8082 | Closed fracture of pubis |
| 8083 | Open fracture of pubis |
| 8084 | Closed fracture of other specified part of pelvis |
| 80841 | Closed fracture of ilium |
| 80842 | Closed fracture of ischium |
| 80843 | Multiple closed pelvic fractures with disruption of pelvic circle |
| 80849 | Closed fracture of other specified part of pelvis |
| 8085 | Open fracture of other specified part of pelvis |
| 80851 | Open fracture of ilium |
| 80852 | Open fracture of ischium |
| 80853 | Multiple open pelvic fractures with disruption of pelvic circle |
| 80854 | Multiple open pelvic fractures without disruption of pelvic circle |
| 80859 | Open fracture of other specified part of pelvis |
| 8088 | Closed unspecified fracture of pelvis |
| 8089 | Open unspecified fracture of pelvis |
| 8090 | Fracture of bones of trunk, closed |
| 810 | Fracture of clavicle |
| 8100 | Closed fracture of clavicle |
| 81000 | Closed fracture of clavicle, unspecified part |
| 81001 | Closed fracture of sternal end of clavicle |
| 81002 | Closed fracture of shaft of clavicle |
| 81003 | Closed fracture of acromial end of clavicle |
| 8101 | Open fracture of clavicle |
| 81010 | Open fracture of clavicle, unspecified part |
| 81011 | Open fracture of sternal end of clavicle |
| 81012 | Open fracture of shaft of clavicle |
| 81013 | Open fracture of acromial end of clavicle |
| 811 | Fracture of scapula |
| 8110 | Closed fracture of scapula |
| 81100 | Closed fracture of scapula, unspecified part |
| 81101 | Closed fracture of acromial process of scapula |
| 81102 | Closed fracture of coracoid process of scapula |
| 81103 | Closed fracture of glenoid cavity and neck of scapula |
| 81109 | Closed fracture of scapula, other |
| 81110 | Open fracture of scapula, unspecified part |
| 81111 | Open fracture of acromial process of scapula |
| 81112 | Open fracture of coracoid process |
| 81113 | Open fracture of glenoid cavity and neck of scapula |
| 81119 | Open fracture of scapula, other |
| 812 | Fracture of humerus |
| 8120 | Fracture of upper end of humerus closed |
| 81200 | Closed fracture of unspecified part of upper end of humerus |
| 81201 | Closed fracture of surgical neck of humerus |
| 81202 | Closed fracture of anatomical neck of humerus |
| 81203 | Closed fracture of greater tuberosity of humerus |
| 81209 | Other closed fracture of upper end of humerus |
| 8121 | Fracture of upper end of humerus open |
| 81210 | Open fracture of unspecified part of upper end of humerus |
| 81211 | Open fracture of surgical neck of humerus |
| 81212 | Open fracture of anatomical neck of humerus |
| 81213 | Open fracture of greater tuberosity of humerus |
| 81219 | Other open fracture of upper end of humerus |
| 8122 | Closed fracture of shaft or unspecified part of humerus |
| 81220 | Closed fracture of unspecified part of humerus |
| 81221 | Closed fracture of shaft of humerus |
| 8123 | Fracture of shaft or unspecified part of humerus open |
| 81230 | Open fracture of unspecified part of humerus |
| 81231 | Open fracture of shaft of humerus |
| 8124 | Fracture of lower end of humerus closed |
| 81240 | Closed fracture of unspecified part of lower end of humerus |
| 81241 | Closed supracondylar fracture of humerus |
| 81242 | Closed fracture of lateral condyle of humerus |
| 81243 | Closed fracture of medial condyle of humerus |
| 81244 | Closed fracture of unspecified condyle(s) of humerus |
| 81249 | Other closed fracture of lower end of humerus |
| 8125 | Fracture of lower end of humerus open |
| 81250 | Open fracture of unspecified part of lower end of humerus |
| 81251 | Open supracondylar fracture of humerus |
| 81252 | Open fracture of lateral condyle of humerus |
| 81253 | Open fracture of medial condyle of humerus |
| 81254 | Open fracture of unspecified condyle(s) of humerus |
| 81259 | Other open fracture of lower end of humerus |
| 813 | Fracture of radius and ulna |
| 8130 | Fracture of upper end of radius and ulna closed |
| 81300 | Closed fracture of upper end of forearm, unspecified |
| 81301 | Closed fracture of olecranon process of ulna |
| 81302 | Closed fracture of coronoid process of ulna |
| 81303 | Closed Monteggia’s fracture |
| 81304 | Other and unspecified closed fractures of proximal end of ulna (alone) |
| 81305 | Closed fracture of head of radius |
| 81306 | Closed fracture of neck of radius |
| 81307 | Other and unspecified closed fractures of proximal end of radius (alone) |
| 81308 | Closed fracture of radius with ulna, upper end [any part] |
| 8131 | Fracture of upper end of radius and ulna open |
| 81310 | Open fracture of upper end of forearm, unspecified |
| 81311 | Open fracture of olecranon process of ulna |
| 81312 | Open fracture of coronoid process of ulna |
| 81313 | Open Monteggia’s fracture |
| 81314 | Other and unspecified open fractures of proximal end of ulna (alone) |
| 81315 | Open fracture of head of radius |
| 81316 | Open fracture of neck of radius |
| 81317 | Other and unspecified open fractures of proximal end of radius (alone) |
| 81318 | Open fracture of radius with ulna, upper end (any part) |
| 8132 | Fracture of shaft of radius and ulna closed |
| 81320 | Closed fracture of shaft of radius or ulna, unspecified |
| 81321 | Closed fracture of shaft of radius (alone) |
| 81322 | Closed fracture of shaft of ulna (alone) |
| 81323 | Closed fracture of shaft of radius with ulna |
| 8133 | Fracture of shaft of radius and ulna open |
| 81330 | Open fracture of shaft of radius or ulna, unspecified |
| 81331 | Open fracture of shaft of radius (alone) |
| 81332 | Open fracture of shaft of ulna (alone) |
| 81333 | Open fracture of shaft of radius with ulna |
| 8134 | Fracture of lower end of radius and ulna closed |
| 81340 | Closed fracture of lower end of forearm, unspecified |
| 81341 | Closed Colles’ fracture |
| 81342 | Other closed fractures of distal end of radius (alone) |
| 81343 | Closed fracture of distal end of ulna (alone) |
| 81344 | Closed fracture of lower end of radius with ulna |
| 81345 | Torus fracture of radius (alone) |
| 81346 | Torus fracture of ulna (alone) |
| 81347 | Torus fracture of radius and ulna |
| 8135 | Fracture of lower end of radius and ulna open |
| 81350 | Open fracture of lower end of forearm, unspecified |
| 81351 | Open Colles’ fracture |
| 81352 | Other open fractures of distal end of radius (alone) |
| 81353 | Open fracture of distal end of ulna (alone) |
| 81354 | Open fracture of lower end of radius with ulna |
| 8138 | Fracture of unspecified part of radius with ulna closed |
| 81380 | Closed fracture of unspecified part of forearm |
| 81381 | Closed fracture of unspecified part of radius (alone) |
| 81382 | Closed fracture of unspecified part of ulna (alone) |
| 81383 | Closed fracture of unspecified part of radius with ulna |
| 8139 | Fracture of unspecified part of radius with ulna open |
| 81390 | Open fracture of unspecified part of forearm |
| 81391 | Open fracture of unspecified part of radius (alone) |
| 814 | Fracture of carpal bone(s) |
| 8140 | Closed fractures of carpal bones |
| 81400 | Closed fracture of carpal bone, unspecified |
| 81401 | Closed fracture of navicular [scaphoid] bone of wrist |
| 81402 | Closed fracture of lunate [semilunar] bone of wrist |
| 81403 | Closed fracture of triquetral [cuneiform] bone of wrist |
| 81404 | Closed fracture of pisiform bone of wrist |
| 81405 | Closed fracture of trapezium bone [larger multangular] of wrist |
| 81406 | Closed fracture of trapezoid bone [smaller multangular] of wrist |
| 81407 | Closed fracture of capitate bone [os magnum] of wrist |
| 81408 | Closed fracture of hamate [unciform] bone of wrist |
| 81409 | Closed fracture of other bone of wrist |
| 81410 | Open fracture of carpal bone, unspecified |
| 81411 | Open fracture of navicular [scaphoid] bone of wrist |
| 81412 | Open fracture of lunate [semilunar] bone of wrist |
| 81413 | Open fracture of triquetral [cuneiform] bone of wrist |
| 81414 | Open fracture of pisiform bone of wrist |
| 81415 | Open fracture of trapezium bone [larger multangular] of wrist |
| 81416 | Open fracture of trapezoid bone [smaller multangular] of wrist |
| 81417 | Open fracture of capitate bone [os magnum] of wrist |
| 81418 | Open fracture of hamate [unciform] bone of wrist |
| 81419 | Open fracture of other bone of wrist |
| 815 | Fracture of metacarpal bone(s) |
| 8150 | Closed fracture of metacarpal bones |
| 81500 | Closed fracture of metacarpal bone(s), site unspecified |
| 81501 | Closed fracture of base of thumb [first] metacarpal |
| 81502 | Closed fracture of base of other metacarpal bone(s) |
| 81503 | Closed fracture of shaft of metacarpal bone(s) |
| 81504 | Closed fracture of neck of metacarpal bone(s) |
| 81509 | Closed fracture of multiple sites of metacarpus |
| 81510 | Open fracture of metacarpal bone(s), site unspecified |
| 81511 | Open fracture of base of thumb [first] metacarpal |
| 81512 | Open fracture of base of other metacarpal bone(s) |
| 81513 | Open fracture of shaft of metacarpal bone(s) |
| 81519 | Open fracture of multiple sites of metacarpus |
| 816 | Fracture of one or more phalanges of hand |
| 8160 | Closed fracture of one or more phalanges of hand |
| 81600 | Closed fracture of phalanx or phalanges of hand, unspecified |
| 81601 | Closed fracture of middle or proximal phalanx or phalanges of hand |
| 81602 | Closed fracture of distal phalanx or phalanges of hand |
| 81603 | Closed fracture of multiple sites of phalanx or phalanges of hand |
| 81610 | Open fracture of phalanx or phalanges of hand, unspecified |
| 81611 | Open fracture of middle or proximal phalanx or phalanges of hand |
| 81612 | Open fracture of distal phalanx or phalanges of hand |
| 81613 | Open fracture of multiple sites of phalanx or phalanges of hand |
| 817 | Multiple closed fractures of hand bones |
| 8170 | Multiple closed fractures of hand bones |
| 8171 | Multiple open fractures of hand bones |
| 818 | Ill-defined fractures of upper limb |
| 8180 | Ill-defined closed fractures of upper limb |
| 8181 | Ill-defined open fractures of upper limb |
| 819 | Multiple fractures involving both upper limbs and upper limb with rib(s) and sternum |
| 8190 | Multiple closed fractures involving both upper limbs, and upper limb with rib(s) and sternum |
| 8191 | Multiple open fractures involving both upper limbs, and upper limb with rib(s) and sternum |
| 820 | Fracture of neck of femur |
| 8200 | Transcervical fracture closed |
| 82000 | Closed fracture of intracapsular section of neck of femur, unspecified |
| 82001 | Closed fracture of epiphysis (separation) (upper) of neck of femur |
| 82002 | Closed fracture of midcervical section of neck of femur |
| 82003 | Closed fracture of base of neck of femur |
| 82009 | Other closed transcervical fracture of neck of femur |
| 8201 | Transcervical fracture open |
| 82010 | Open fracture of intracapsular section of neck of femur, unspecified |
| 82011 | Open fracture of epiphysis (separation) (upper) of neck of femur |
| 82012 | Open fracture of midcervical section of neck of femur |
| 8202 | Pertrochanteric fracture of femur closed |
| 82020 | Closed fracture of trochanteric section of neck of femur |
| 82021 | Closed fracture of intertrochanteric section of neck of femur |
| 82022 | Closed fracture of subtrochanteric section of neck of femur |
| 8203 | Pertrochanteric fracture of femur open |
| 82030 | Open fracture of trochanteric section of neck of femur, unspecified |
| 82031 | Open fracture of intertrochanteric section of neck of femur |
| 82032 | Open fracture of subtrochanteric section of neck of femur |
| 8208 | Closed fracture of unspecified part of neck or femur |
| 8209 | Open fracture of unspecified part of neck of femur |
| 821 | Fracture of other and unspecified parts of femur |
| 8210 | Fracture of shaft or unspecified part of femur closed |
| 82100 | Closed fracture of unspecified part of femur |
| 82101 | Closed fracture of shaft of femur |
| 82103 | Open fracture of base of neck of femur |
| 82109 | Other open transcervical fracture of neck of femur |
| 8211 | Fracture of shaft or unspecified part of femur open |
| 82110 | Open fracture of unspecified part of femur |
| 82111 | Open fracture of shaft of femur |
| 8212 | Fracture of lower end of femur closed |
| 82120 | Closed fracture of lower end of femur, unspecified part |
| 82121 | Closed fracture of condyle, femoral |
| 82122 | Closed fracture of epiphysis, lower (separation) of femur |
| 82123 | Closed supracondylar fracture of femur |
| 82129 | Other closed fracture of lower end of femur |
| 8213 | Fracture of lower end of femur open |
| 82130 | Other fracture of lower end of femur, unspecified part |
| 82131 | Other fracture of condyle, femoral |
| 822 | Fracture of patella |
| 8220 | Closed fracture of patella |
| 8221 | Open fracture of patella |
| 823 | Fracture of tibia and fibula |
| 8230 | Fracture of upper end of tibia and fibula closed |
| 82300 | Closed fracture of upper end of tibia alone |
| 82301 | Closed fracture of upper end of fibula alone |
| 82302 | Closed fracture of upper end of fibula with tibia |
| 8231 | Fracture of upper end of tibia and fibula open |
| 82310 | Open fracture of upper end of tibia alone |
| 82311 | Open fracture of upper end of fibula alone |
| 82312 | Open fracture of upper end of fibula with tibia |
| 8232 | Fracture of shaft of tibia and fibula closed |
| 82320 | Closed fracture of shaft of tibia alone |
| 82321 | Closed fracture of shaft of fibula alone |
| 82322 | Closed fracture of shaft of fibula with tibia |
| 8233 | Fracture of tibia and fibula |
| 82330 | Open fracture of shaft of tibia alone |
| 82331 | Open fracture of shaft of fibula alone |
| 82332 | Open fracture of shaft and fibula with tibia |
| 8234 | Fracture of tibia and fibula, torus fracture |
| 82340 | Torus fracture, tibia alone |
| 82341 | Torus fracture, fibula alone |
| 82342 | Torus fracture, fibula with tibia |
| 8238 | Fracture of unspecified part of tibia and fibula closed |
| 82380 | Closed fracture of unspecified part of tibia alone |
| 82381 | Closed fracture of unspecified part of fibula alone |
| 82382 | Closed fracture of unspecified part of fibula with tibia |
| 8239 | Fracture of unspecified part of tibia and fibula open |
| 82390 | Open fracture of unspecified part of tibia alone |
| 82391 | Open fracture of unspecified part of fibula alone |
| 82392 | Open fracture of unspecified part of fibula with tibia |
| 824 | Fracture of ankle |
| 8240 | Fracture of medial malleolus, closed |
| 8241 | Fracture of medial malleolus, open |
| 8242 | Fracture of lateral malleolus, closed |
| 8243 | Fracture of lateral malleolus, open |
| 8244 | Bimalleolar fracture, closed |
| 8245 | Bimalleolar fracture, open |
| 8246 | Trimalleolar fracture, closed |
| 8247 | Trimalleolar fracture, open |
| 8248 | Unspecified fracture of ankle, closed |
| 8249 | Unspecified fracture of ankle, open |
| 825 | Fracture of one or more tarsal and metatarsal bones |
| 8250 | Fracture of calcaneus, closed |
| 8251 | Fracture of calcaneus, open |
| 8252 | Fracture of other tarsal and metatarsal bones closed |
| 82520 | Closed fracture of unspecified bone(s) of foot [except toes] |
| 82521 | Closed fracture of astragalus |
| 82522 | Closed fracture of navicular [scaphoid], foot |
| 82523 | Closed fracture of cuboid |
| 82524 | Closed fracture of cuneiform, foot |
| 82525 | Closed fracture of metatarsal bone(s) |
| 82529 | Other closed fracture of tarsal and metatarsal bones |
| 8253 | Fracture of other tarsal and metatarsal bones open |
| 82530 | Open fracture of unspecified bone(s) of foot [except toes] |
| 82531 | Open fracture of astragalus |
| 82532 | Open fracture of navicular [scaphoid], foot |
| 82533 | Open fracture of cuboid |
| 82534 | Open fracture of cuneiform, foot |
| 82535 | Open fracture of metatarsal bone(s) |
| 82539 | Other open fracture of tarsal and metatarsal bones |
| 826 | Fracture of one or more phalanges of foot |
| 8260 | Closed fracture of one or more phalanges of foot |
| 8261 | Open fracture of one or more phalanges of foot |
| 827 | Other multiple and ill-defined fractures of lower limb |
| 8271 | Other, multiple and ill-defined fractures of lower limb, closed |
| 8272 | Other, multiple and ill-defined fractures of lower limb, open |
| 828 | Multiple fractures involving both lower limbs lower with upper limb and lower limb(s) with rib(s) and sternum |
| 8280 | Closed multiple fractures involving both lower limbs, lower with upper limb, and lower limb(s) with rib(s) and sternum |
| 8281 | Open multiple fractures involving both lower limbs, lower with upper limb, and lower limb(s) with rib(s) and sternum |
| 829 | Fracture of unspecified bones |
| 8290 | Fracture of unspecified bone, closed |
| 8291 | Fracture of unspecified bone, open |
| E887 | Fracture, cause unspecified |

Table S-2. Codes to identify other skeletal-related events (radiation therapy, surgery on bone, and spinal cord compression)

| Type | Code | | Description |
| --- | --- | --- | --- |
| **Procedure codes to identify radiation therapy** | | | |
| ICD-9 | 9223 | | Radioisotopic teleradiotherapy |
| ICD-9 | 9224 | | Teleradiotherapy using photons |
| ICD-9 | 9229 | | Other radiotherapeutic procedure |
| ICD-9 | 9230 | | Stereotactic radiosurgery, not otherwise specified |
| ICD-9 | 9231 | | Single source photon radiosurgery |
| ICD-9 | 9232 | | Multi-source photon radiosurgery |
| ICD-9 | 9239 | | Stereotactic radiosurgery, not elsewhere classified |
| HCPCS | A9600 | | Strontium sr-89 chloride, therapeutic, per millicurie |
| HCPCS | A9604 | | Samarium sm-153 lexidronam, therapeutic, per treatment dose, up to 150 millicuries |
| HCPCS | A9605 | | Samarium sm-153 lexidronam, therapeutic, per 50 millicuries |
| HCPCS | C9401 | | Supply of therapeutic radiopharmaceutical, strontium-89 chloride, brand name, per mci |
| HCPCS | G0173 | | Linear accelerator–based stereotactic radiosurgery, complete course of therapy in one session |
| HCPCS | G0174 | | Intensity modulated radiation therapy delivery |
| HCPCS | G0243 | | Multi-source photon stereotactic radiosurgery, delivery including collimator changes and custom plugging, complete course of treatment, all lesions |
| HCPCS | G0251 | | Linear accelerator–based stereotactic radiosurgery, delivery including collimator changes and custom plugging, fractionated treatment, all lesions, per session, maximum five sessions per course of treatment |
| HCPCS | G0339 | | Image-guided, robotic, linear accelerator–based stereotactic radiosurgery; complete course of therapy in one session or first session of fractionated treatment |
| HCPCS | G0340 | | Image-guided, robotic, linear accelerator–based stereotactic radiosurgery; delivery including collimator changes and custom plugging, fractionated treatment, all lesions, per session, second through fifth sessions, maximum five sessions per course of treatment |
| HCPCS | J3005 | | Injection, strontium-89 chloride, per 10 ml |
| HCPCS | 0073T | | Compensator-based beam modulation treatment delivery of inverse planned treatment using three or more high resolution (milled or cast) compensator‑convergent beam-modulated fields per treatment session |
| HCPCS | 61793 | | Stereotactic radiosurgery (particle beam, gamma ray or linear accelerator), one or more sessions |
| HCPCS | 61796 | | Stereotactic radiosurgery (particle beam, gamma ray, or linear accelerator); 1 simple cranial lesion |
| HCPCS | 61797 | | Stereotactic radiosurgery (particle beam, gamma ray, or linear accelerator), each additional cranial lesion, simple |
| HCPCS | 61798 | | Stereotactic radiosurgery (particle beam, gamma ray, or linear accelerator); 1 complex cranial lesion |
| HCPCS | 63620 | | Stereotactic radiosurgery (particle beam, gamma ray, or linear accelerator); 1 spinal lesion |
| HCPCS | 63621 | | Stereotactic radiosurgery (particle beam, gamma ray, or linear accelerator); each additional spinal lesion |
| HCPCS | 77371 | | Radiation treatment delivery, stereotactic radiosurgery, complete course of treatment of cranial lesion(s) consisting of one session; multi-source Cobalt 60 based |
| HCPCS | 77372 | | Radiation treatment delivery, stereotactic radiosurgery, complete course of treatment of cranial lesion(s) consisting of one session; linear accelerator based |
| HCPCS | 77373 | | Stereotactic body radiation therapy, treatment delivery, per fraction to one or more lesions, including image guidance, entire course not to exceed five fractions |
| HCPCS | 77401 | | Radiation treatment delivery, superficial and/or ortho voltage, per day |
| HCPCS | 77402 | | Radiation treatment delivery, ≥1 MeV; simple |
| HCPCS | 77403 | | Radiation treatment delivery; single treatment area, single port or parallel opposed ports, simple blocks or no blocks; 11-19 MeV |
| HCPCS | 77404 | | Radiation treatment delivery; single treatment area, single port or parallel opposed ports, simple blocks or no blocks; 6-10 MeV |
| HCPCS | 77407 | | Radiation treatment delivery, ≥1 MeV; intermediate |
| HCPCS | 77408 | | Radiation treatment delivery, 2 separate treatment areas, 3 or more ports on a single treatment area, use of multiple blocks; 6-10 MeV |
| HCPCS | 77409 | | Radiation treatment delivery, 2 separate treatment areas, 3 or more ports on a single treatment area, use of multiple blocks; 11-19 MeV |
| HCPCS | 77411 | | Radiation treatment delivery; two separate treatment areas, three or more ports on a single treatment area use of multiple blocks; 20 MeV or greater |
| HCPCS | 77412 | | Radiation treatment delivery, ≥1 MeV; complex |
| HCPCS | 77413 | | Radiation treatment delivery, 3 or more separate treatment areas, custom blocking, tangential ports, wedges, rotational beam, compensators, electron beam; 6-10 MeV |
| HCPCS | 77414 | | Radiation treatment delivery, 3 or more separate treatment areas, custom blocking, tangential ports, wedges, rotational beam, compensators, electron beam; 11-19 MeV |
| HCPCS | 77416 | | Radiation treatment delivery, 3 or more separate treatment areas, custom blocking, tangential ports, wedges, rotational beam, compensators, electron beam; 20 MeV or greater |
| HCPCS | 77418 | | Intensity modulated treatment delivery, single or multiple fields/arcs, via narrow spatially and temporally modulated beams, binary, dynamic multileaf collimator, per treatment session |
| HCPCS | 79005 | | Radiopharmaceutical therapy, by oral administration |
| HCPCS | 79101 | | Radiopharmaceutical therapy, by intravenous administration |
| HCPCS | 79200 | | Radiopharmaceutical therapy, by intracavitary administration |
| HCPCS | 79300 | | Radiopharmaceutical therapy, by interstitial radioactive colloid administration |
| HCPCS | 79400 | | Radiopharmaceutical therapy, nonthyroid, nonhematologic by intervenous injection |
| HCPCS | 79403 | | Radiopharmaceutical therapy, radiolabeled monoclonal antibody by intravenous infusion |
| HCPCS | 79440 | | Radiopharmaceutical therapy, by intra-articular administration |
| HCPCS | 79445 | | Radiopharmaceutical therapy, by intra-arterial particulate administration |
| HCPCS | 79999 | | Radiopharmaceutical therapy, unlisted procedure |
| **Procedure codes to identify surgery on bone** | | | |
| Type | | Code | Description |
| ICD-9 | | 7815 | Application of external fixator device, femur |
| ICD-9 | | 7845 | Other repair or plastic operations on bone, femur |
| ICD-9 | | 7855 | Internal fixation of bone without fracture reduction, femur |
| ICD-9 | | 7915 | Closed reduction of fracture with internal fixation, femur |
| ICD-9 | | 7925 | Open reduction of fracture without internal fixation, femur |
| ICD-9 | | 7935 | Open reduction of fracture with internal fixation, femur |
| ICD-9 | | 7995 | Unspecified operation on bone injury, femur |
| ICD-9 | | 7812 | Application of external fixator device, humerus |
| ICD-9 | | 7842 | Other repair or plastic operations on bone, humerus |
| ICD-9 | | 7852 | Internal fixation of bone without fracture reduction, humerus |
| ICD-9 | | 7911 | Closed reduction of fracture with internal fixation, humerus |
| ICD-9 | | 7921 | Open reduction of fracture without internal fixation, humerus |
| ICD-9 | | 7931 | Open reduction of fracture with internal fixation, humerus |
| ICD-9 | | 7991 | Unspecified operation on bone injury, humerus |
| ICD-9 | | 7813 | Application of external fixator device, radius and ulna |
| ICD-9 | | 7843 | Other repair or plastic operations on bone, radius and ulna |
| ICD-9 | | 7853 | Internal fixation of bone without fracture reduction, radius and ulna |
| ICD-9 | | 7912 | Closed reduction of fracture with internal fixation, radius and ulna |
| ICD-9 | | 7922 | Open reduction of fracture without internal fixation, radius and ulna |
| ICD-9 | | 7932 | Open reduction of fracture with internal fixation, radius and ulna |
| ICD-9 | | 7992 | Unspecified operation on bone injury, radius and ulna |
| ICD-9 | | 7817 | Application of external fixator device, tibia and fibula |
| ICD-9 | | 7847 | Other repair or plastic operations on bone, tibia and fibula |
| ICD-9 | | 7857 | Internal fixation of bone without fracture reduction, tibia and fibula |
| ICD-9 | | 7916 | Closed reduction of fracture with internal fixation, tibia and fibula |
| ICD-9 | | 7926 | Open reduction of fracture without internal fixation, tibia and fibula |
| ICD-9 | | 7936 | Open reduction of fracture with internal fixation, tibia and fibula |
| ICD-9 | | 7996 | Unspecified operation on bone injury, tibia and fibula |
| ICD-9 | | 0353 | Repair of vertebral fracture |
| ICD-9 | | 8102 | Other cervical fusion of the anterior column, anterior technique |
| ICD-9 | | 8103 | Other cervical fusion of the posterior column, posterior technique |
| ICD-9 | | 8104 | Dorsal and dorsolumbar fusion of the anterior column, anterior technique |
| ICD-9 | | 8105 | Dorsal and dorsolumbar fusion of the posterior column, posterior technique |
| ICD-9 | | 8106 | Lumbar and lumbosacral fusion of the anterior column, anterior technique |
| ICD-9 | | 8107 | Lumbar and lumbosacral fusion of the posterior column, posterior technique |
| ICD-9 | | 8108 | Lumbar and lumbosacral fusion of the anterior column, posterior technique |
| ICD-9 | | 7810 | Application of external fixator device, unspecified site |
| ICD-9 | | 7816 | Application of external fixator device, patella |
| ICD-9 | | 7819 | Application of external fixator device, other bones |
| ICD-9 | | 7840 | Other repair or plastic operations on bone, unspecified site |
| ICD-9 | | 7841 | Other repair or plastic operations on bone, scapula, clavicle, and thorax [ribs and sternum] |
| ICD-9 | | 7846 | Other repair or plastic operations on bone, patella |
| ICD-9 | | 7849 | Other repair or plastic operations on bone, other bones |
| ICD-9 | | 7850 | Internal fixation of bone without fracture reduction, unspecified site |
| ICD-9 | | 7851 | Internal fixation of bone without fracture reduction, scapula, clavicle, and thorax [ribs and sternum] |
| ICD-9 | | 7856 | Internal fixation of bone without fracture reduction, patella |
| ICD-9 | | 7859 | Internal fixation of bone without fracture reduction, other bones |
| ICD-9 | | 7910 | Closed reduction of fracture with internal fixation, unspecified site |
| ICD-9 | | 7919 | Closed reduction of fracture with internal fixation, other specified bone |
| ICD-9 | | 7920 | Open reduction of fracture without internal fixation, unspecified site |
| ICD-9 | | 7929 | Open reduction of fracture without internal fixation, other specified bone |
| ICD-9 | | 7930 | Open reduction of fracture with internal fixation, unspecified site |
| ICD-9 | | 7939 | Open reduction of fracture with internal fixation, other specified bone |
| ICD-9 | | 7990 | Unspecified operation on bone injury, unspecified site |
| ICD-9 | | 7999 | Unspecified operation on bone injury, other specified bone |
| CPT | | 27187 | Prophylactic treatment (nailing, pinning, plating, or wiring) with or without methylmethacrylate, femoral neck and proximal femur |
| CPT | | 27235 | Percutaneous skeletal fixation of femoral fracture, proximal end, neck |
| CPT | | 27236 | Open treatment of femoral fracture, proximal end, neck, internal fixation or prosthetic replacement |
| CPT | | 27244 | Treatment of intertrochanteric, peritrochanteric, or subtrochanteric femoral fracture; with plate/screw type implant, with or without cerclage |
| CPT | | 27245 | Treatment of intertrochanteric, peritrochanteric, or subtrochanteric femoral fracture; with intramedullary implant, with or without interlocking screws and/or cerclage |
| CPT | | 27248 | Open treatment of greater trochanteric fracture, includes internal fixation, when performed |
| CPT | | 27269 | Open treatment of femoral fracture, proximal end, head, includes internal fixation when performed |
| CPT | | 27495 | Prophylactic treatment (nailing, pinning, plating, or wiring) with or without methylmethacrylate, femur |
| CPT | | 27506 | Open treatment of femoral shaft fracture, with or without external fixation, with insertion of intramedullary implant, with or without cerclage and/or locking screws |
| CPT | | 27507 | Open treatment of femoral shaft fracture with plate/screws, with or without cerclage |
| CPT | | 27509 | Percutaneous skeletal fixation of femoral fracture, distal end, medial or lateral condyle, or supracondylar or transcondylar, with or without intercondylar extension, or distal femoral epiphyseal separation |
| CPT | | 27511 | Open treatment of femoral supracondylar or transcondylar fracture without intercondylar extension, includes internal fixation, when performed |
| CPT | | 27513 | Open treatment of femoral supracondylar or transcondylar fracture with intercondylar extension, includes internal fixation when performed |
| CPT | | 27514 | Open treatment of femoral fracture, distal end, medial or lateral condyle, includes internal fixation when performed |
| CPT | | 23615 | Open treatment of proximal humeral (surgical or anatomical neck) fracture, includes internal fixation, when performed, includes repair of tuberosity(s), when performed |
| CPT | | 23616 | Open treatment of proximal humeral (surgical or anatomical neck) fracture, includes internal fixation, when performed, includes repair of tuberosity(s), when performed; with proximal humeral prosthetic replacement |
| CPT | | 23630 | Open treatment of greater humeral tuberosity fracture, includes internal fixation, when performed |
| CPT | | 24498 | Prophylactic treatment (nailing, pinning, plating or wiring), with or without methylmethacrylate, humeral shaft |
| CPT | | 24515 | Open treatment of humeral shaft fracture with plate/screws, with or without cerclage |
| CPT | | 24516 | Treatment of humeral shaft fracture, with insertion of intramedullary implant, with or without cerclage and/or locking screws |
| CPT | | 24538 | Percutaneous skeletal fixation of supracondylar or transcondylar humeral fracture, with or without intercondylar extension |
| CPT | | 24545 | Open treatment of humeral supracondylar or transcondylar fracture, includes internal fixation, when performed; without intercondylar extension |
| CPT | | 24546 | Open treatment of humeral supracondylar or transcondylar fracture, includes internal fixation, when performed; with intercondylar extension |
| CPT | | 24566 | Percutaneous skeletal fixation of humeral epicondylar fracture, medial or lateral, with manipulation |
| CPT | | 24575 | Open treatment of humeral epicondylar fracture, medial or lateral, includes internal fixation, when performed |
| CPT | | 24579 | Open treatment of humeral condylar fracture, medial or lateral, includes internal fixation, when performed |
| CPT | | 24582 | Percutaneous skeletal fixation of humeral condylar fracture, medial or lateral, with manipulation |
| CPT | | 24586 | Open treatment of periarticular fracture and/or dislocation of the elbow (fracture distal humerus and proximal ulna and/or proximal radius) |
| CPT | | 24587 | Open treatment of periarticular fracture and/or dislocation of the elbow (fracture distal humerus and proximal ulna and/or proximal radius); with implant arthroplasty |
| CPT | | 24635 | Open treatment of Monteggia type of fracture dislocation at elbow (fracture proximal end of ulna with dislocation of radial head), includes internal fixation, when performed |
| CPT | | 24665 | Open treatment of radial head or neck fracture, includes internal fixation r radial head excision, when performed |
| CPT | | 24666 | Open treatment of radial head or neck fracture, includes internal fixation r radial head excision, when performed; with radial head prosthetic treatment |
| CPT | | 24685 | Open treatment of ulnar fracture, proximal end (e.g., olecranon or coronoid process[es]), includes internal fixation, when performed |
| CPT | | 25490 | Prophylactic treatment (nailing, pinning, plating or wiring), with or without methylmethacrylate; radius |
| CPT | | 25491 | Prophylactic treatment (nailing, pinning, plating or wiring), with or without methylmethacrylate; ulna |
| CPT | | 25492 | Prophylactic treatment (nailing, pinning, plating or wiring), with or without methylmethacrylate; radius and ulna |
| CPT | | 25515 | Open treatment of radial shaft fracture, includes internal fixation, when performed |
| CPT | | 25525 | Open treatment of radial shaft fracture, includes internal fixation, when performed, and closed treatment of distal radioulnar joint dislocation (Galeazzi fracture/dislocation), includes percutaneous skeletal fixation, when performed |
| CPT | | 25526 | Open treatment of radial shaft fracture, includes internal fixation, when performed, and open treatment of distal radioulnar joint dislocation (Galeazzi fracture/dislocation), includes internal fixation, when performed, includes repair of triangular fibrocartilage complex |
| CPT | | 25545 | Open treatment of ulnar shaft fracture, includes internal fixation, when performed |
| CPT | | 25606 | Percutaneous skeletal fixation of distal radial fracture or epiphyseal separation |
| CPT | | 25607 | Open treatment of distal radial extra-articular fracture or epiphyseal separation, with internal fixation |
| CPT | | 25608 | Open treatment of distal radial intra-articular fracture or epiphyseal separation; with internal fixation of two fragments |
| CPT | | 25609 | Open treatment of distal radial intra-articular fracture or epiphyseal separation; with internal fixation of three or more fragments |
| CPT | | 27535 | Open treatment of tibial fracture, proximal (plateau); unicondylar, includes internal fixation, when performed |
| CPT | | 27536 | Open treatment of tibial fracture, proximal (plateau); bicondylar, with or without internal fixation |
| CPT | | 27745 | Prophylactic treatment (nailing, pinning, plating, or wiring) with or without methylmethacrylate, tibia |
| CPT | | 27756 | Percutaneous skeletal fixation of tibial shaft fracture (with or without fibular fracture) (e.g., pin or screws) |
| CPT | | 27758 | Open treatment of tibial shaft fracture (with or without fibular fracture), with plate/screws, with or without cerclage |
| CPT | | 27759 | Treatment of tibial shaft fracture (with or without fibular fracture) by intramedullary implant, with or without interlocking screws and/or cerclage |
| CPT | | 27766 | Open treatment of medial malleolus fracture, includes internal fixation, when performed |
| CPT | | 27769 | Open treatment of posterior malleolus fracture, includes internal fixation, when performed |
| CPT | | 27784 | Open treatment of proximal fibula or shaft fracture; includes internal fixation, when performed |
| CPT | | 27792 | Open treatment of distal fibular fracture (lateral malleolus), includes internal fixation, when performed |
| CPT | | 27826 | Open treatment of fracture of weight bearing articular surface/portion of distal tibia (e.g., pilon or tibial plafond), with internal fixation, when performed; of fibula only |
| CPT | | 27827 | Open treatment of fracture of weight bearing articular surface/portion of distal tibia (e.g., pilon or tibial plafond), with internal fixation, when performed; of tibia only |
| CPT | | 22325 | Open treatment and/or reduction of vertebral fracture(s) and/or dislocation(s), posterior approach, 1 fractured vertebra or dislocated segment; lumbar |
| CPT | | 22326 | Open treatment and/or reduction of vertebral fracture(s) and/or dislocation(s), posterior approach, 1 fractured vertebra or dislocated segment; cervical |
| CPT | | 22327 | Open treatment and/or reduction of vertebral fracture(s) and/or dislocation(s), posterior approach, 1 fractured vertebra or dislocated segment; thoracic |
| CPT | | 22328 | Open treatment and/or reduction of vertebral fracture(s) and/or dislocation(s), posterior approach, 1 fractured vertebra or dislocated segment; each additional fractured vertebra or dislocated segment (List separately in addition to code for primary procedure) |
| CPT | | 22520 | Percutaneous vertebroplasty (bone biopsy included when performed), 1 vertebral body, unilateral or bilateral injection; thoracic |
| CPT | | 22521 | Percutaneous vertebroplasty (bone biopsy included when performed), 1 vertebral body, unilateral or bilateral injection; lumbar |
| CPT | | 22522 | Percutaneous vertebroplasty (bone biopsy included when performed), 1 vertebral body, unilateral or bilateral injection; each additional thoracic or lumbar vertebral body (List separately in addition to code for primary procedure) |
| CPT | | 22532 | Arthrodesis, lateral extracavitary technique, including minimal discectomy to prepare interspace (other than for decompression); thoracic |
| CPT | | 22533 | Arthrodesis, lateral extracavitary technique, including minimal discectomy to prepare interspace (other than for decompression); lumbar |
| CPT | | 22534 | Arthrodesis, lateral extracavitary technique, including minimal discectomy to prepare interspace (other than for decompression); thoracic or lumbar, each additional vertebral segment (List separately in addition to code for primary procedure) |
| CPT | | 22548 | Arthrodesis, anterior transoral or extraoral technique, clivus-C1-C2 (atlas-axis), with or without excision of odontoid process |
| CPT | | 22554 | Arthrodesis, anterior interbody technique, including minimal discectomy to prepare interspace (other than for decompression); cervical below C2 |
| CPT | | 22556 | Arthrodesis, anterior interbody technique, including minimal discectomy to prepare interspace (other than for decompression); thoracic |
| CPT | | 22558 | Arthrodesis, anterior interbody technique, including minimal discectomy to prepare interspace (other than for decompression); lumbar |
| CPT | | 22585 | Arthrodesis, anterior interbody technique, including minimal discectomy to prepare interspace (other than for decompression); each additional interspace (List separately in addition to code for primary procedure) |
| CPT | | 22590 | Arthrodesis, posterior technique, craniocervical (occiput-C2) |
| CPT | | 22595 | Arthrodesis, posterior technique, atlas-axis (C1-C2) |
| CPT | | 22600 | Arthrodesis, posterior or posterolateral technique, single level; cervical below C2 segment |
| CPT | | 22610 | Arthrodesis, posterior or posterolateral technique, single level; thoracic (with lateral transverse technique, when performed) |
| CPT | | 22612 | Arthrodesis, posterior or posterolateral technique, single level; lumbar (with lateral transverse technique, when performed) |
| CPT | | 22614 | Arthrodesis, posterior or posterolateral technique, single level; each additional vertebral segment (List separately in addition to code for primary procedure) |
| CPT | | 22630 | Arthrodesis, posterior interbody technique, including laminectomy and/or discectomy to prepare interspace (other than for decompression), single interspace; lumbar |
| CPT | | 22632 | Arthrodesis, posterior interbody technique, including laminectomy and/or discectomy to prepare interspace (other than for decompression), single interspace; each additional interspace (List separately in addition to code for primary procedure) |
| CPT | | 20982 | Ablation, bone tumor(s) (e.g., osteoid osteoma metastasis radiofrequency) percutaneous, including computer tomographic guidance |
| CPT | | 23490 | Prophylactic treatment (nailing, pinning, plating or wiring) with or without methylmethacrylate; clavicle |
| CPT | | 23515 | Open treatment of clavicular fracture, includes internal fixation, when performed |
| CPT | | 23585 | Open treatment of scapular fracture (body, glenoid or acromion) includes internal fixation, when performed |
| CPT | | 27215 | Open treatment of iliac spine(s), tuberosity avulsion, or iliac wing fracture(s), unilateral, for pelvic bone fracture patterns that do not disrupt the pelvic ring, includes internal fixation, when performed |
| CPT | | 27216 | Percutaneous skeletal fixation of posterior pelvic bone fracture and/or dislocation, for fracture patterns that disrupt the pelvic ring, unilateral (includes ipsilateral ilium, sacroiliac joint and/or sacrum) |
| CPT | | 27217 | Open treatment of anterior pelvic bone fracture and/or dislocation for fracture patterns that disrupt the pelvic ring, unilateral, includes internal fixation, when performed (includes pubic symphysis and/or ipsilateral superior/inferior rami) |
| CPT | | 27218 | Open treatment of posterior pelvic bone fracture and/or dislocation, for fracture patterns that disrupt the pelvic ring, unilateral, includes internal fixation, when performed (includes ipsilateral ilium, sacroiliac joint and/or sacrum) |
| CPT | | 27226 | Open treatment of posterior or anterior acetabular wall fracture, with internal fixation |
| CPT | | 27227 | Open treatment of acetabular fracture(s) involving anterior or posterior (one) column, or a fracture running transversely across the acetabulum, with internal fixation |
| CPT | | 27228 | Open treatment of acetabular fracture(s) involving anterior and posterior (two) columns, includes T-fracture and both column fracture with complete articular detachment, or single column or transverse fracture with associated acetabular wall fracture, with internal fixation |
| CPT | | 27524 | Open treatment of patellar fracture, with internal fixation and/or partial or complete patellectomy and soft tissue repair |
| CPT | | 27540 | Open treatment of intercondylar spine(s) and/or tuberosity fracture(s) of the knee, includes internal fixation, when performed |
| CPT | | 22523 | Percutaneous vertebral augmentation, including cavity creation (fracture reduction and bone biopsy included when performed) using mechanical device, 1 vertebral body, unilateral or bilateral cannulation (e.g., kyphoplasty); thoracic |
| CPT | | 22524 | Percutaneous vertebral augmentation, including cavity creation (fracture reduction and bone biopsy included when performed) using mechanical device, 1 vertebral body, unilateral or bilateral cannulation (e.g., kyphoplasty); lumbar |
| CPT | | 22525 | Percutaneous vertebral augmentation, including cavity creation (fracture reduction and bone biopsy included when performed) using mechanical device, 1 vertebral body, unilateral or bilateral cannulation (e.g., kyphoplasty); each additional thoracic or lumbar vertebral body (List separately in addition to code for primary procedure) |
| CPT | | 22526 | Percutaneous intradiscal electrothermal annuloplasty, unilateral or bilateral including fluoroscopic guidance; single level |
| CPT | | 22527 | Percutaneous intradiscal electrothermal annuloplasty, unilateral or bilateral including fluoroscopic guidance; one or more additional levels |
| CPT | | 25574 | Open treatment of radial AND ulnar shaft fractures, with internal fixation, when performed; of radius OR ulna |
| CPT | | 25575 | Open treatment of radial AND ulnar shaft fractures, with internal fixation, when performed; of radius and ulna |

CPT = Current Procedural Terminology; HCPCS = Healthcare Common Procedure Coding System; ICD-9 = *International Classification of Diseases, 9th Revision.*
